# Achieving nano-gold stability through rational design†
†Electronic supplementary information (ESI) available. See DOI: 10.1039/c6sc01597b



**DOI:** 10.1039/c6sc01597b

**Published:** 2016-07-20

**Authors:** Dean H. Barrett, Michael S. Scurrell, Cristiane B. Rodella, Beatriz Diaz, David G. Billing, Paul J. Franklyn

**Affiliations:** a Molecular Sciences Institute , School of Chemistry , University of the Witwatersrand , Private Bag PO Wits , Braamfontein , 2050 , South Africa . Email: paul.franklyn@wits.ac.za; b Brazilian Synchrotron Light Laboratory (LNLS)/Brazilian Center for Research in Energy and Materials (CNPEM) , C. P. 6192 , 13083-970 , Campinas , SP , Brazil . Email: dean.barrett@lnls.br; c Dept. of Civil & Chemical Engineering , University of South Africa , Johannesburg , Florida 1710 , South Africa; d Canadian Light Source , 44 Innovation Boulevard , Saskatoon , SK S7N 2V3 , Canada; e DST-NRF Centre of Excellence in Strong Materials , School of Chemistry , University of the Witwatersrand , Private Bag PO Wits , Braamfontein , 2050 , South Africa

## Abstract

When Au is subdivided to the nanoscale its reactivity changes from an inert nature to one of incredible reactivity which is not replicated by other catalysts. When dispersed onto metal oxides such as TiO_2_, nano-Au has shown high reactivities for a multitude of reduction and oxidation reactions of industrial importance with potential and current uses such as, CO oxidation, NO_*x*_ reduction, purification of hydrogen for fuel cells, water gas shift reactions, abatement of volatile organic compounds (VOC's) as well as pollution and emission control systems such as autocatalysts. However, many industrially important reactions and applications operate under harsh conditions where the catalyst is exposed to high temperatures and further needs to operate for extended periods of time. These conditions cause Au nanoparticle sintering whereby small, highly active clusters form large clusters which are catalytically inactive. For this reason, research into stabilizing Au nanoparticles has abounded with a goal of producing durable, thermally stable catalysts for industrial applications. Here we show a durable, thermally stable Au–TiO_2_ catalyst which has been developed by rational design. The catalyst exhibits a 3-dimensional, radially aligned nanorod structure, already locked into the thermodynamically stable polymorph, *via* a scalable and facile synthesis, with Au nanoparticles isolated on the support structure. As the Au nanoparticles are highly stable the new catalyst is able to maintain light-off for CO oxidation below 115 °C even after multiple cycles at 800 °C. This ability of the catalyst to resist multiple thermal cycles to high temperature while remaining active at low temperatures shows promise for various industrial applications. The thermal stability of the catalyst is investigated and characterized through morphological and structural studies.

## Abstract 2

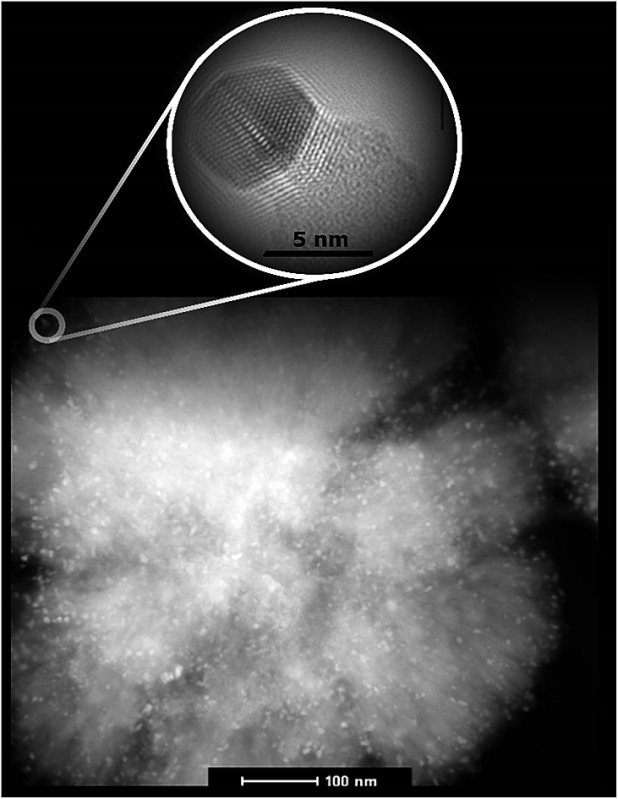
Au nanoparticles isolated on the tips of a thermally stable, radially aligned nano-structure providing a highly sinter-resistant catalyst.

Heterogeneous catalysis makes use of nanoparticulate materials to produce chemicals and materials which we rely on in everyday life with applications from fuels, pharmaceuticals, polymers to pollution reduction amongst others.^1^ However, sintering and the consequent loss of surface area and active sites results in these valuable catalysts deactivating to a point where they no longer function and need to be replaced at great financial cost.

In 1989 Haruta *et al.* reported that supported Au nanoparticles are highly active in the catalytic oxidation of carbon monoxide.^2^ Fourteen years later, in a review for Science, Adrian Cho wrote: “For Au nanoparticles to become truly practical however, researchers must overcome the particles' inherent tendency to clump together especially when heated or exposed to reactant gasses”.^3^ This is because only small, highly dispersed Au nanoparticles exhibit exceptional catalytic activity at low temperature.^3^

If Au nanoparticles can be stabilized a number of potential applications become viable.^5–8^ For example, most pollution from automobiles is emitted in the first 5 minutes after start-up, while the engine is still being warmed. This is because platinum group metals that constitute the active phase of current automotive catalysts are poisoned by CO at lower temperatures. For this reason, light-off, the temperature at which the catalytic reaction is initiated, in current catalytic converters occurs between 200 and 300 °C resulting in light-off pollution.^9^ Nano-Au catalysts, however, light-off well below these temperatures but deactivate rapidly due to the poor thermal stability of nano-Au as the catalytic activity is strongly dependent on Au particle size for many reactions.^3–12^

Various modification techniques have been applied to supported Au catalysts in attempts to reduce Au sintering.^13,14^ The composition of the catalyst support is an important factor and the best results are usually obtained using TiO_2_.^15–20^ Au and TiO_2_ operate in a synergic manner with active sites located at the perimeter interface between them, where O_2_ adsorbs preferentially and dissociates.^15–20^ Thus, a sinter resistant catalyst is crucial to retain small Au nanoparticles, which preserve these highly active perimeter sites.

Scanning tunneling microscopy (STM) and density functional theory (DFT) studies have shown that substrate defects of TiO_2_ play a major role in the stabilization of Au nanoparticles.^21–23^ Further, Au nanoparticles prefer to nucleate at surface defects such as oxygen vacancies (unsaturated Ti ions) and step edges. The surface defects of TiO_2_ go as far as determining the shape and distribution of Au nanoparticles over the surface. Defect sites on TiO_2_ are produced under a variety of conditions in both oxidative and reducing atmospheres. The reduction of TiO_2_ by hydrogen not only results in oxygen vacancy formation but also creates Ti interstitials in the TiO_2_ matrix.^24^ Even under oxygen-rich conditions, oxygen vacancies are favoured over interstitials. Under oxygen-poor conditions, both defect types are stabilized, with the number of Ti interstitials predicted to increase in rutile phase as a function of temperature.^24,25^ These defects directly affect the chemical and physical properties of TiO_2_ which in turn affect the mobility of Au particles over the surface.^26,27^

For Au supported on rutile (110), Schaub *et al.* showed that as temperature increases the decrease in the number of oxygen vacancies is considerably larger due to Au clusters being trapped over multiple oxygen vacancies, with Au clusters bound to these oxygen vacancy sites.^28^ With further heating, more growth occurs and single oxygen vacancies can no longer stabilize the larger clusters, resulting in the cluster-vacancy complex diffusing. While diffusing the cluster can encounter either another cluster (leading to coalescence) or other surface defects. In the former case, a larger cluster is formed which itself may continue to diffuse. However, if the cluster-vacancy complex encounters other vacancies, it can bind to them and, therefore, stabilize on the surface. Step edges themselves are stabilizing positions for the larger Au clusters, as they can be considered as a collection of oxygen vacancies.^28^

Nanostructures with high curvature play an important role in the formation of undercoordinated Ti sites, which act in the same manner as defect sites. Rabatic *et al.* ^29^ conducted TEM investigations of surface defect sites present at the tips of high-aspect-ratio ellipsoid nanoparticles of TiO_2_. They showed that specific undercoordinated “corner” defect sites that constitute the surface of high-curvature nanoparticles are responsible for the chemical modification of nanostructured TiO_2_. Fast Fourier transforms were performed at the bulk and tip areas of the anisotropic particles, revealing significant differences in the lattice structure of the respective regions. An EPR investigation of TiO_2_ nanorods showed strong localization of electrons at high curvature sites (*i.e.*, tips).^30^ The findings show the nanorod tips impart an undercoordinated atomic character of Ti sites which provide further defects which would act to stabilize Au nanoparticles. In summary, support surfaces, which provide high numbers of defect sites (oxygen vacancies, step edges and Ti interstitials), more effectively reduce the mobility of Au nanoparticles over their surface which reduces sintering.

Barring a few incremental improvements over the last decades, the emergence of a sinter resistant, nano-Au catalyst with stable Au nanoparticles has not come to fruition. Here we show a new catalyst which provides nano-Au stability in a definitive, reproducible and generally applicable way.^31^

## Results and discussion

### Deactivation studies

Commercially available Aurolite catalyst® (1% Au–TiO_2_ (P25 Degussa)), as well as a 5% Au–TiO_2_ (P25 Degussa), referred to from now as commercial Au–TiO_2_, were used as baseline standards to assess the thermal stability of Au catalysts over long durations (<50 h). *In situ* powder X-ray diffraction (PXRD) measurements were performed from room temperature to 810 °C under air to study Au sintering. The phase transition from metastable anatase to thermodynamically stable rutile provoked intense Au-nanoparticle sintering (Fig. S6†). These results in combination with transmission electron microscopy (TEM), Brunauer–Emmett–Teller (BET) surface area analysis and catalytic testing showed that structural instability of the support is a major factor in Au-nanoparticle growth. All data, as well as its discussion, can be accessed in the ESI.† The outcomes of the aforementioned studies were applied to the rational design of a new catalyst, involving key functions which were advertently synthesized according to the intended function of the new catalyst.

### Rational design

**Fig. 1:**
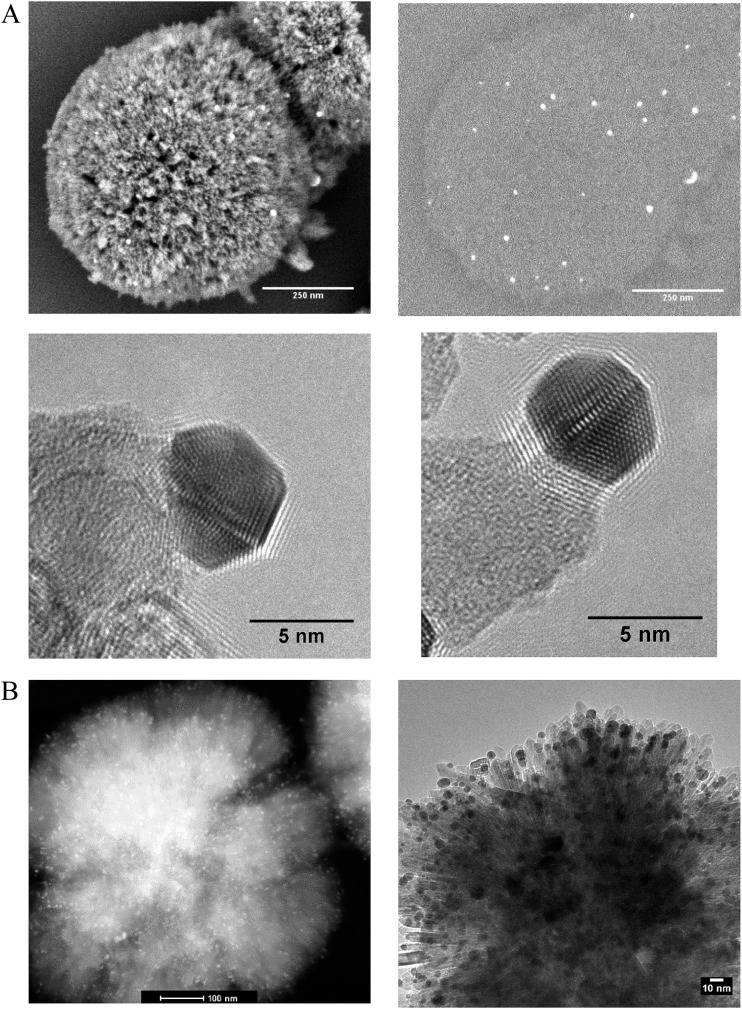
(A) SEM image (top left) of the catalyst after activation with the corresponding BSE image (top right) highlighting the Au nanoparticles as bright flashes. The images show the dispersion of Au nanoparticles which are isolated on the periphery of the structure after activation of the catalyst under hydrogen. HRTEM (bottom left and right) after activation of the catalyst. The images show the well-defined structure and distinct surface geometry of the Au nanoparticles at the tips of the nanorods as well as showing evidence of strong binding between the titania and the Au nanoparticles, (B) (Left) HAADF image of catalyst confirming the location of the Au (bright white spots) at the tips of the rods and showing the morphology of the RANR after *in situ* PXRD measurements under synthetic air. (Right) TEM image of the catalyst after *in situ* PXRD measurements for over 200 hours and holding at 810 °C for 5 hours confirmed minimal growth of the nano-Au.

Phase pure titanium dioxide rutile nanorods with a specific morphology referred to as radially aligned nano rutile or RANR was prepared using a mild hydrothermal synthesis at ambient pressure**.** Alignment of the rutile nanorods was verified by electron diffraction, TEM (Fig. 1B) and *in situ* PXRD (Fig. S9†) with each nanorod having the same crystallographic orientation. The RANR consistently shows surface areas around 100 m^2^ g^–1^ providing relatively high usable surface area.

Nanoparticulate Au was deposited onto the RANR with theoretical loadings of 1.2%, 5% and 6% Au by mass using deposition–precipitation with urea (Sl). Rietveld refinement and pair distribution function (PDF) analysis confirmed Au loadings of 1%, 3.7% and 5.4%, respectively (Fig. S10A–C and S16–S21 respectively†).

**Fig. 2:**
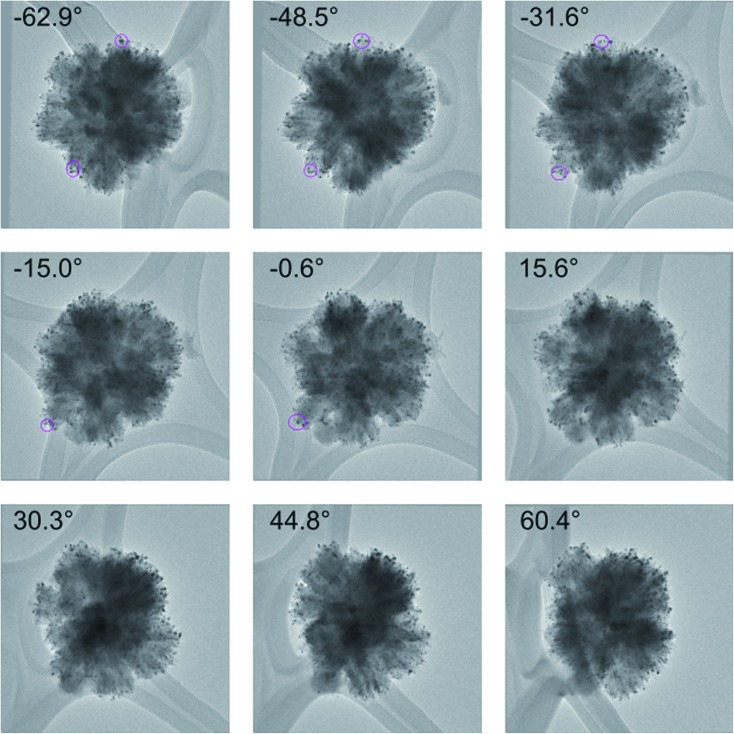
A selection of images used for the TEM tilt series of the 5% Au-RANR catalyst after *in situ* PXRD measurements. Selected Au nanoparticles were tracked using fiducial tracking during the rotation to confirm their positions. The reconstruction video can be accessed in the ESI.†

Scanning electron microscopy (SEM), back scatter SEM (BSE), high angle annular dark field (HAADF), TEM, and TEM tomography confirmed the presence of the Au nanoparticles isolated on the nanorods both before and after thermal treatments (*in situ* PXRD) under synthetic air to 810 °C (Fig. 1A and B and 2)**.** Prior to thermal treatment, the catalysts were activated under hydrogen flow at 300 °C for 1 hour. A TEM tilt series (Fig. 2) conducted after the *in situ* PXRD data collection (heating for over 200 h and holding at 810 °C for 5 h) confirmed the minimal growth of the Au and RANR structure. A TEM tilt series (Fig. 2) conducted after thermal treatment of the catalyst was used to track the location of the Au nanoparticles for confirmation that Au was not located within the structure but remained highly dispersed and isolated even after high-temperature exposure (Tomo video available in ESI†).

The RANR structure differs from conventional supports in various ways, and especially, with respect to the morphology. The RANR structure consists of radial nanorods with high aspect ratio (Fig. 1 and 2). Not only do the tips of the nanorods provide strained, curved surfaces but the cylindrical nature of the nanorods also provides curvature, albeit to a lesser degree. Thus, the assembly of the RANR nanorods in an anisotropic fashion provide enhanced defect sites, which aid Au stability. The presence of Au nanoparticles at the tips of the RANR, as shown by TEM tomography, confirms this finding. Undercoordinated defect sites located on the tips of the nanorods act as binding sites for the Au nanoparticles, effectively isolating them at the tips of the structure even after thermal treatment to 810 °C.^29^

### *In situ* PXRD

The verification of the Au nanoparticle stability was completed through long duration *in situ* PXRD data collections as well as PDF analysis and catalytic testing during thermal cycling. Long duration *in situ* PXRD data collection for Au-RANR (Fig. S9 and S12†) was conducted (>200 h). Thus, the catalysts were exposed to temperatures of 550–810 °C for over 140 h. Diffractograms show 100% rutile (TiO_2_) phase composition for Au-RANR, with anisotropic, broad diffraction peaks due to the catalysts nanostructure properties as well as the common alignment of the nanorods (Fig. S9 and S10A–C†).

**Fig. 3:**
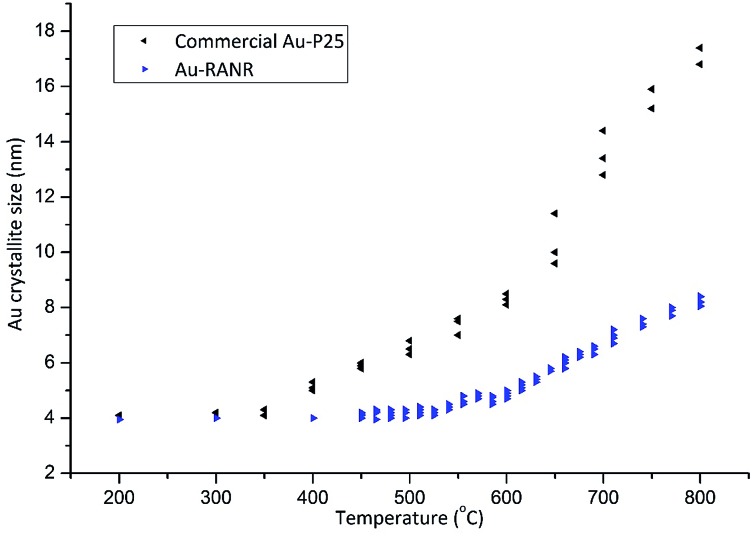
Au crystallite sizes of the commercial Au–TiO_2_ and Au-RANR catalysts determined from Rietveld refinement from *in situ* PXRD.

The diffractograms (Fig. S9†) confirmed the moderate anisotropic growth of the rutile nanorods seen in TEM (Fig. 1A and B and 2). Rietveld analysis of the *in situ* PXRD data collection provided quantitative information about the catalysts’ structures as the temperature was increased. The Au crystallite sizes of the commercial Au–TiO_2_ and Au-RANR catalysts are graphed *versus* temperature in Fig. 3.

The TEM size distributions (Fig. S15†) of Au nanoparticles after thermal treatment to 810 °C are centered around 6.5 nm. Rietveld refinements performed on the X-ray diffraction data gave average Au crystallite sizes of 8.0 ± 0.3 nm, which corresponds well with TEM results (Fig. S15†). This result confirms that no large Au particles are present within the structure as XRD probes the bulk of the sample. With the majority of Au nanoparticles maintaining their small size, highly active perimeter sites are retained. The slow and gradual increase in the Au nanoparticle size during heating of the Au-RANR catalyst can in part be attributed to small morphological changes in the support structure (Fig. 1). The nanorods extrude and broaden slightly over the temperature range, confirmed by *in situ* PXRD and TEM, and correspond to a small loss in surface determined by BET with a loss of only 20% of its original surface area (97 to 77 m^2^ g^–1^).

Comparisons between the RANR support with and without Au show interesting results. When Au is deposited onto the RANR, the growth of the structure during thermal treatment is inhibited as shown in Fig. S11.† This is in contrast to the commercial catalysts, where the presence of Au-catalyzed the phase transformation and destabilized the catalyst (Fig. S5 and S6†).

The presence of thermodynamically unstable phases of TiO_2_ leads to large structural changes and associated loss of surface area resulting in sintering and encapsulation of active sites (Fig. S5 and S6†). Phase purity is, therefore, essential in order to avoid anatase or other unstable phases of TiO_2_ converting under non-ambient conditions. As the Au-RANR catalyst is comprised of thermodynamically stable rutile, no phase transition is possible. The morphology and high aspect ratio of the Au-RANR is thus maintained even after thermal cycling as seen by TEM and *in situ* PXRD (Fig. 1, S9 and S10A–C†). The retention of surface area and high aspect ratio combined with thermodynamic phase stability with increased numbers of defect sites effectively stabilize the Au nanoparticles. The RANR structure further aids Au stability at high temperatures, when particle migration is more likely as only a limited number of Au particles are present on each nanorod. Some Au sintering does occur to a small extent, as seen by *in situ* PXRD and TEM. However, once this occurs, the Au nanoparticles thereafter remain isolated over the numerous, individual nanorods which exhibit terminating crystal planes with defect type undercoordinated Ti on the strained tips. Therefore, it is proposed that the combination of the physical separation, arising from the isolation of the Au nanoparticles towards the tips of the nanorods, and advantageous surface chemistry helps to maintain the position of the Au nanoparticles on the peripheries, preventing particle growth.

Pair-distribution function (PDF) measurements were conducted *in situ* with measurements of the Au-RANR structure again showing controlled growth from the reduction of the Au species to metallic Au at 200 °C and during further heating (Fig. S16–S21†), confirming the results from *in situ* PXRD.

### Catalytic cycling

**Fig. 4:**
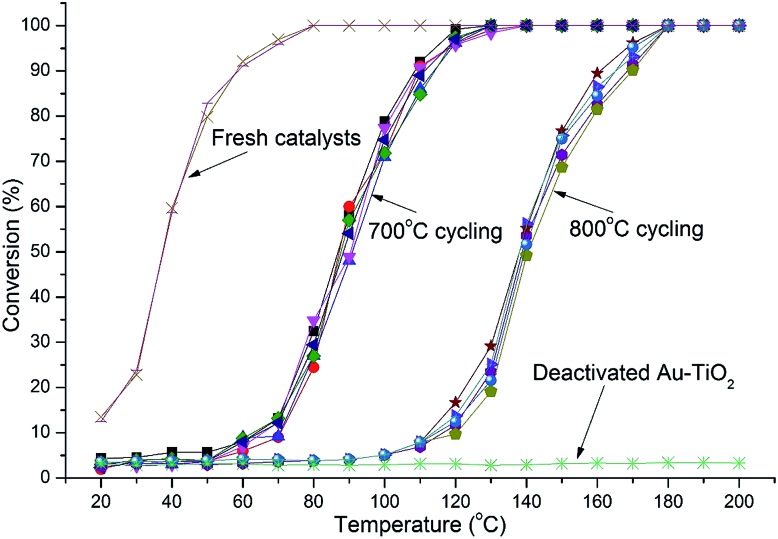
Light-off curves for catalysts with 1.2% Au-RANR and commercial Au–TiO_2_ after multiple 700 and 800 °C heating cycles (10 cycles in total).

**Fig. 5:**
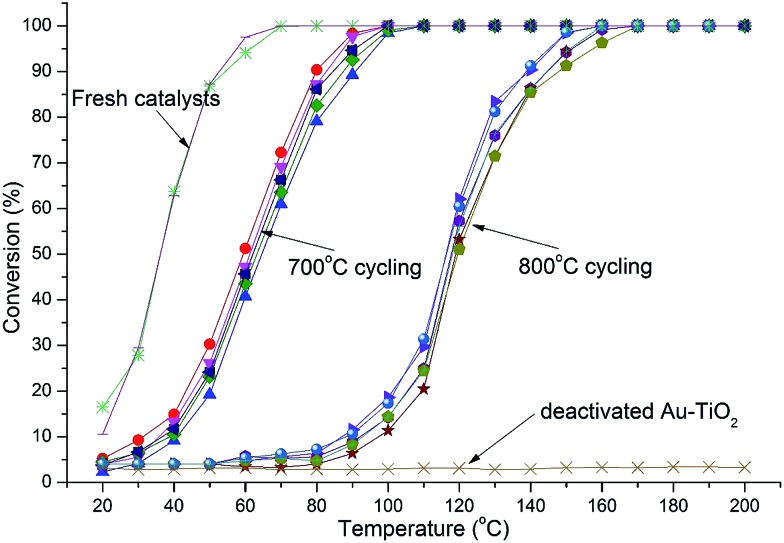
Light-off curves for catalysts with 5% Au-RANR and commercial Au–TiO_2_ catalyst after multiple 700 and 800 °C heating cycles (10 cycles in total).

Catalytic testing was undertaken with the use of the CO oxidation reaction. The reaction served two purposes. Firstly, CO oxidation is an industrially important reaction in various applications and secondly, CO oxidation is highly structure sensitive with respect to the Au nanoparticle size.^3,4^ Thus, the reaction acts as an ideal probe to aid in characterizing the catalyst *via* catalytic data. Thermal cycling under harsh conditions, long duration, high temperature, oxidizing atmosphere, and multiple cycles is important to determine a catalysts durability. Catalysts were therefore thermally cycled to 700 °C, 5 times using a heating rate of 8 °C min^–1^ and then held isothermally for 3 h before being cooled to 20 °C. Light-off curves were then measured and the catalyst was cycled again. After 4 cycles the catalysts were again heated to 700 °C but held isothermally for 12 h, cooled and then light-off curves attained. Thermal cycling was then conducted using the same catalyst to 800 °C in the same manner as before comprising 4 cycles to 800 °C and finally a 12 h isothermal treatment at 800 °C. Samples comprised 1.2 and 5% Au loadings by mass. Heating cycles were conducted under oxygen-rich atmosphere as this has been shown to enhance sintering of Au nanoparticles.^32^ The results of catalytic tests are shown in Fig. 4 and 5.

Both Au-RANR and commercial Au–TiO_2_ initially show high activity with light-off temperatures from 20 °C, as they contain well dispersed, small Au-nanoparticles able to readily catalyse the reaction (Fig. S1, S8†, 4 and 5). As temperatures increased the well-documented effect of substantial Au sintering takes place for the commercial Au–TiO_2_ catalysts resulting in complete deactivation after a short period of thermal exposure (1 cycle at 700 °C),^10–14^ ESI.†

The 1.2% Au-RANR catalyst shows T_50_ temperatures around 80 and 135 °C for the 700 and 800 °C tests, respectively (Fig. 4). *T*_50_ temperatures around 60 and 115 °C were observed for 5% Au-RANR catalysts (Fig. 5). Cycling of the catalysts indicates that an initial loss of activity occurs during the first thermal treatment, as the catalyst adjusts to reduce high surface energies with an initial particle migration occurring on the nanorods.^28^ However, once this process occurs the catalyst can then be cycled numerous times, without further loss of activity. This effect is shown in the difference between light-off temperatures of catalysts cycled at 700 and then 800 °C with a shift of approximately 50 °C in the light-off temperatures (Fig. 4 and 5). The turnover frequencies (TOF's) for the 1.2% Au loaded catalyst were determined at 30 °C for both fresh and cycled catalysts to 800 °C. TOF's were calculated from the surface exposed Au atoms determined from both size and geometric considerations. 0.29 s^–1^ was attained by the fresh catalysts with 0.1 s^–1^ for catalyst after thermal cycling. The results show that the catalyst is able to function over a wide temperature range.

While higher Au loadings may provide more active sites they also increase the probability of the loss of active sites *via* sintering at high temperatures as there are more Au species per unit area over the support surface. Increased sintering probability with higher Au loadings is compounded further when the surface area and morphology of the support decrease significantly during thermal treatment placing Au clusters in closer proximity to each other.

**Fig. 6:**
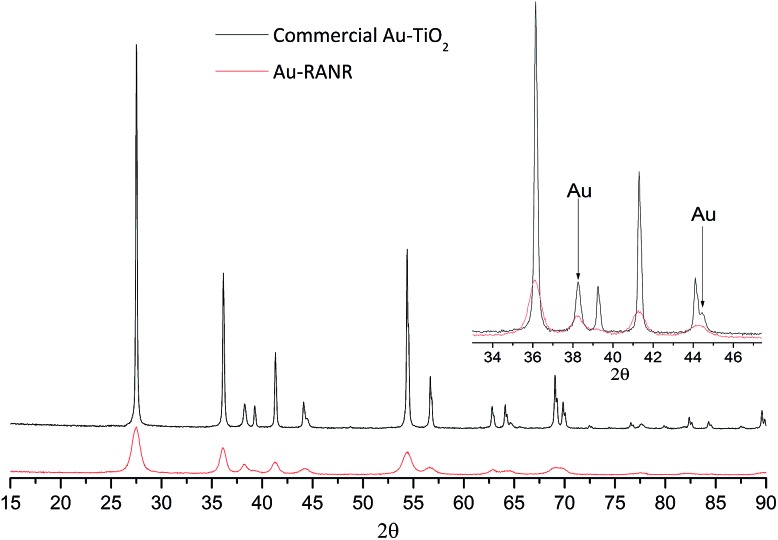
PXRD patterns of 5% Au-RANR and commercial Au–TiO_2_ after thermal treatment during catalytic cycling to 800 °C. Diffractograms of 1.2% Au loadings are shown in Fig. S12 and S13.†

The Au-RANR catalyst overcomes these problems and shows an increased stability with increased Au loading as shown by the lower light-off temperatures after thermal cycling. The radially aligned rutile nanorods provide a beneficial morphology, able to lock relatively high Au loadings on the surface even at high temperature. As rutile is thermodynamically stable no significant transformation of the catalyst occurs during thermal cycling no matter how many times the catalyst is cycled helping retain the surface area and morphology of the catalyst. Diffractograms comparing the commercial Au–TiO_2_ and Au-RANR catalysts after catalytic reaction are shown in Fig. 6.

The overlaid diffractograms (Fig. 6) show the large structural differences between the two catalysts with significantly sharper and more intense Au diffraction peaks for the commercial Au catalysts. Rietveld refinement yields a Au particle size distribution around 7.9 nm for the Au-RANR and 18 nm for commercial Au–TiO_2_. The Au nanoparticle sizes compare well with the *in situ* PXRD and TEM data. Catalytic testing and subsequent XRD analysis of the catalyst reaffirm the stability of the Au nanoparticles on the RANR structure. The highly structure sensitive CO oxidation reaction is still initiated well below the 150 °C mark even after multiple, long duration cycles of 700 and 800 °C.

## Conclusions

To date, the instability of supported nano-Au catalysts has prevented their use in important applications as many reactions are highly structure sensitive to the Au nanoparticle size. Examination of the literature made it clear that an alternate approach was required to stabilize the Au nanoparticles. Now, a thermodynamically stable support material with designed morphology onto which Au nanoparticles are deposited and isolated to reduce mobility across the surface of the catalyst has been demonstrated. HRTEM, HAADF, SEM, TEM tomography, long duration *in situ* PXRD, PDF as well as CO oxidation reactions confirmed the Au-RANR catalysts remarkable structural, thermal and catalytic stability. Further, the Au serves to stabilize the morphology in a mutually beneficial interaction. The catalyst was cycled to 800 °C repeatedly but showed almost no loss of activity after an initial stabilization due to minimal sintering of the Au nanoparticles. The new catalyst shows potential for industrial applications requiring durability and high thermal stability. In addition, the catalyst maintains CO light-off temperatures well below that of any current platinum group metal based catalyst even after rigorous thermal cycling at 800 °C.

## Methods

### Synthesis

Radially aligned nano rutile (RANR) was synthesized *via* a hydrothermal reaction performed under ambient pressure. 12.0 mL of TiCl_4_ was slowly added to 160 mL of deionized water at 0 °C in a round bottomed flask. The solution was heated to 160 °C for 16 h under reflux whilst undergoing rapid stirring. The white precipitate was centrifuged and washed multiple times with hot deionized water to remove all excess Cl. The RANR was then placed into a heating oven and dried overnight at 110 °C. A 250 mL conical flask was placed onto a temperature controlled heater-stirrer and 100 mL of deionized water was added to the conical flask. 1 g of RANR or commercial P25 TiO_2_ was added to the deionized water. HAuCl_4_ : 3H_2_O and 0.85 M urea was also added. The theoretical loading of Au was between 1.2 and 6% depending on the sample. A stirrer bar was added to the conical flask followed by the flask being sealed with the reaction undertaken in a dark fume cupboard so that no light could enter during the synthesis. The solution was heated to 75 °C and 80 °C to ensure the complete hydrolysis of urea. The reaction took place over 24 h. Once the reaction was complete the solution containing the catalyst was washed multiple times with hot deionized water and then centrifuged. This process was repeated until the pH reached neutrality. Finally, the catalyst was then placed in a drying oven overnight at 110 °C to remove any residual water. Reduction was only undertaken just prior to catalyst use at 300 °C for 1 hour under hydrogen flow. Thermal treatments, including *in situ* PXRD measurements and thermal cycling, were then conducted under oxidizing conditions using synthetic air.

### BET

The N_2_ adsorption–desorption experiment was conducted at –193 °C using a Micrometrics TriStar surface area and porosity analyser. Prior to the experiment, the sample was out gassed at 200 °C for 6 h. The BET surface areas were obtained over a relative pressure range from 0.05 to 0.30. The total pore volume was calculated from the amount of N_2_ vapour adsorbed at a relative pressure of 0.99.

### *In situ* PXRD and Rietveld refinements

A Bruker D8 Advance X-ray diffractometer that is equipped with an Anton Paar XRK 900 reaction chamber was used. The diffractometer uses a sealed tube Cu X-ray source and is equipped with a primary beam Göbel mirror and a Bruker VÅNTEC-1 PSD detector using a radial Söller slit and a secondary beam nickel filter. The diffractometer was operated in Bragg–Brentano geometry. XRD measurements were also conducted at the XPD beamline at the LNLS synchrotron in flat plate geometry at a beamline energy of 8 keV with a Dectris Mythen detector.

Quantitative Rietveld refinements were conducted using TOPAS V4.2 in order to determine the relative phase abundances and average crystallite sizes of the crystalline phases present in the sample. Several variables employed in the refinement varied smoothly with temperature or remained constant over the entire temperature range. Therefore, for these refinements, several variables were described using overall descriptions. These include an overall simple axial model to describe axial divergence and an overall parameter to describe the zero-point error of the diffractometer. These variables were averaged over all diffractograms in the data set thus lowering the error in the minimization procedure. Furthermore, several variables which vary independently with temperature were refined. These include lattice parameters of all phases present in the sample as well as the general position of the oxygen atoms within the rutile (monoclinic-TiO_2_) crystal structure. Since the morphology of the sample can be well defined as rod-like for the Au-RANR catalysts it was also deemed necessary to refine several spherical harmonics vectors so as to account for preferred orientation within the sample.

As the background was a convolution of both sample and instrument specific aberrations *i.e.* incoherent scattering and destructive interference which is likely considering that many of the samples are nano-sized. The defined independent variables for each of the two oxygen general positions within the rutile crystal structure are denoted as oxy*XX* where *XX* was a number corresponding to the diffraction pattern number within the dataset. Separately defined variables for each spherical harmonic function over all the data for the rutile structure were imputed.

In the case of the rutile nanorods to account for the direction of propagation of the nanorod structure, the use of the spherical harmonic algorithm was valid. Furthermore, due to the morphology of the nanorods, it was noted that the intensity of the (110) reflection was overestimated while that of the (011) and (111) reflections were underestimated which was noted from the initial fit. In some refinements, the range of the refinement was limited in order to determine the morphology of the nanorods.

### PDF analysis

Measurements were performed at 11-ID-B beamline at the Advanced Photon Source at 58 keV. The samples were placed in capillaries in a reaction cell to control temperature and gas flow during the thermal treatments.^33^ During the experiments, the samples were heated from ambient temperature to 600 °C. A Perkin Elmer detector with 2048x2048 pixels of 200 × 200 μm^2^ was used at 170 mm from the sample in order to measure the scattered radiation to high *q* values. The programs FIT2D, PDFgetX3 and PDFgui were used to convert the detector images to one-dimensional *q vs.* intensity measurements, retrieve the *G*(*r*), and fit the *G*(*r*) to a model including rutile and Au phases.

### Electron microscopy

Data was collected on a Tecnai F20 X-Twin TEM with Cs of 1.0 mm and a point to point resolution limit of 1.9 Å operated at 200 kV. The machine is equipped with a high angle annular dark field (HAADF) detector for scanning transmission electron microscopy, a Gatan electron energy-loss spectroscopy (EELS) filter, and GIF imaging system. The standard bright field imaging system is a 4k chip (2048 × 2048 pixels). Images were collected in spot-size 1 with beam spread to ensure low-dose conditions at 200 kV. Images were collected at, or as close to, the Sherzer defocus setting of the microscope. Samples were loaded onto holey carbon films for analysis. The energy dispersive spectroscopy (EDS) system is an Oxford EDAX system. An FEI Tecnai G2 Spirit electron microscope operating at 120 kV was also used. ImageJ software was used to examine the collected TEM images. TomoJ was used to produce a 3D tomogram of 134 TEM images. SEM images were collected using a Helios NanoLab 660 operating in SEI mode at 2 kV.

### Catalytic testing

50 mg of catalyst was mixed with 250 mg of crushed quartz and placed into a 6 mm internal diameter quartz reactor. A thermocouple was placed inside the capillary adjacent to the catalyst to accurately control temperature during heating as well as during reactions. 2.5% CO/He and 7% O_2_/He was used to provide a modest excess of O_2_ with a GHSV of 63 600 ml h^–1^ g^–1^.

Catalysts were initially heated under a flow of hydrogen at a heating rate of 8 °C per minute to 300 °C and held isothermally for 1 hour. Following the initial heating, gas composition was then changed to synthetic air for all the thermal treatments. Once the thermal treatment was completed the catalyst was cooled to 20 °C and the reaction gas flowed. Measurements were made at 10 °C intervals while 30 minutes stabilization time was given per interval to achieve steady state conditions.

For cycling tests, the catalysts were initially heated to 700 °C at 8 °C per minute where after the catalysts were held isothermally for 3 h and subsequently cooled down to 20 °C to be tested again. This process was repeated 4 times to determine changes in activity over multiple cycles. Finally, the catalysts were heated to the respective temperatures for 12 h and then cooled again for catalytic testing. Following these cycles, the same method was applied but with heating up to 800 °C. Gasses were bubbled through water at 0–1 °C.

Conversion was calculated using the formula CO conversion = CO_in_ – CO_out_/CO_in_ × (100%)

An Agilent 490 micro GC comprising 4 channels and a TCD detector was used for catalytic testing. The system contains two MS5A columns of different length as well as a PPQ and 5CB column. Both MS5A and PPQ columns were used to analyse CO and CO_2_ concentrations.
