# Supplementary material for: Achieving nano-gold stability through rational design
Source: Chem Sci. 2016 Jul 20;7(11):6815–23. doi: 10.1039/c6sc01597b (PMC5134759; doi:10.1039/c6sc01597b)
Supplement: Supplementary file 1 [file SC-007-C6SC01597B-s001.pdf]

Electronic Supplementary Material (ESI) for Chemical Science.
This journal is © The Royal Society of Chemistry 2016

Supplementary information

Achieving nano-gold stability through rational design

Dean H. Barrett,* Michael S. Scurrell, Cristiane B. Rodella, Beatriz Diaz, David G. Billing, and Paul J. Franklyn*

Catalytic testing

Catalytic benchmark testing, commercial 1% Au-P25 (Aurolite®) and 5% Au – P25

For comparative means, a current Au-TiO_2 catalyst was used or order to benchmark current standards for nano-Au catalysts. The Aurolite® catalyst is currently the only commercially produced Au-TiO_2 catalyst comprising of 1% Au (by mass) loaded on P25 type TiO_2 comprising approximately 20% rutile and 80% anatase. A second batch of catalysts was examined where the catalyst composition was similar to that of Aurolite® except with a higher Au loading of 5% in order to test the effect of higher Au loadings on catalytic activity. Catalysts were thermally cycled to 700°C and then 800°C, however, the catalytic testing was shortened as the catalysts deactivated after only one thermal cycle to 700°C. The results of the catalytic tests are shown below in Figure S1.

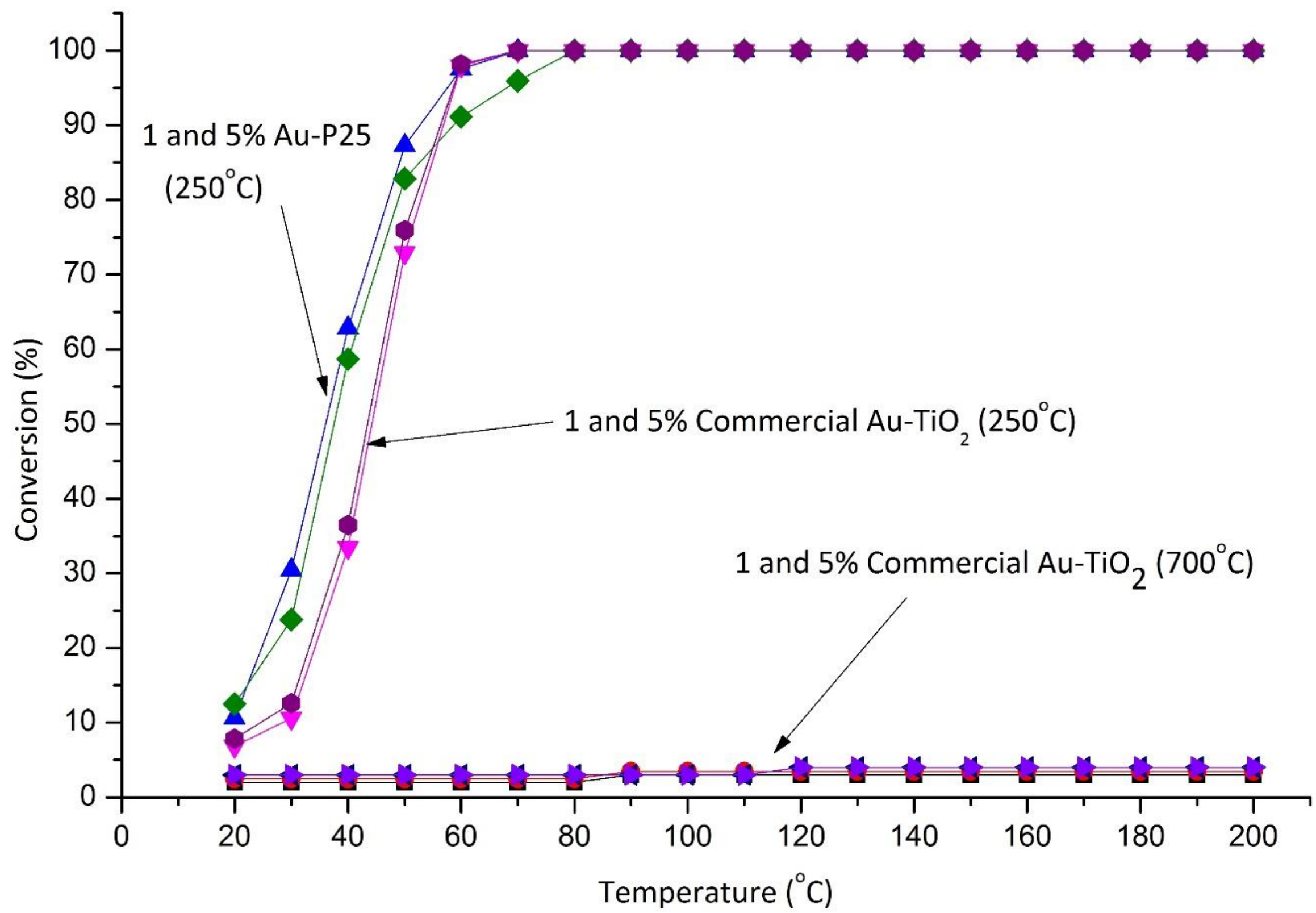

Figure S1: Catalytic tests of Aurolite® (1% Au-P25) and 5% Au-P25

The results of the catalytic tests showed highly active catalysts for both fresh and 250°C samples with T_{50} light-off temperatures of the catalysts around 35°C. However, after a thermal cycle to 700°C complete deactivation of the catalysts occurred. Even at a reaction temperature of 200°C, the catalysts showed no catalytic activity. An investigation into the structural changes of the catalyst which result in deactivation was undertaken beginning with *in-situ* powder X-ray diffraction studies.

In-situ PXRD

Long duration *in-situ* diffraction studies were conducted over a period of 54 h whereby the catalyst was heated from ambient temperature to 810°C using a heating rate of 5°C/min in air to simulate a rigorous thermal treatment as shown in Figure S2 below.

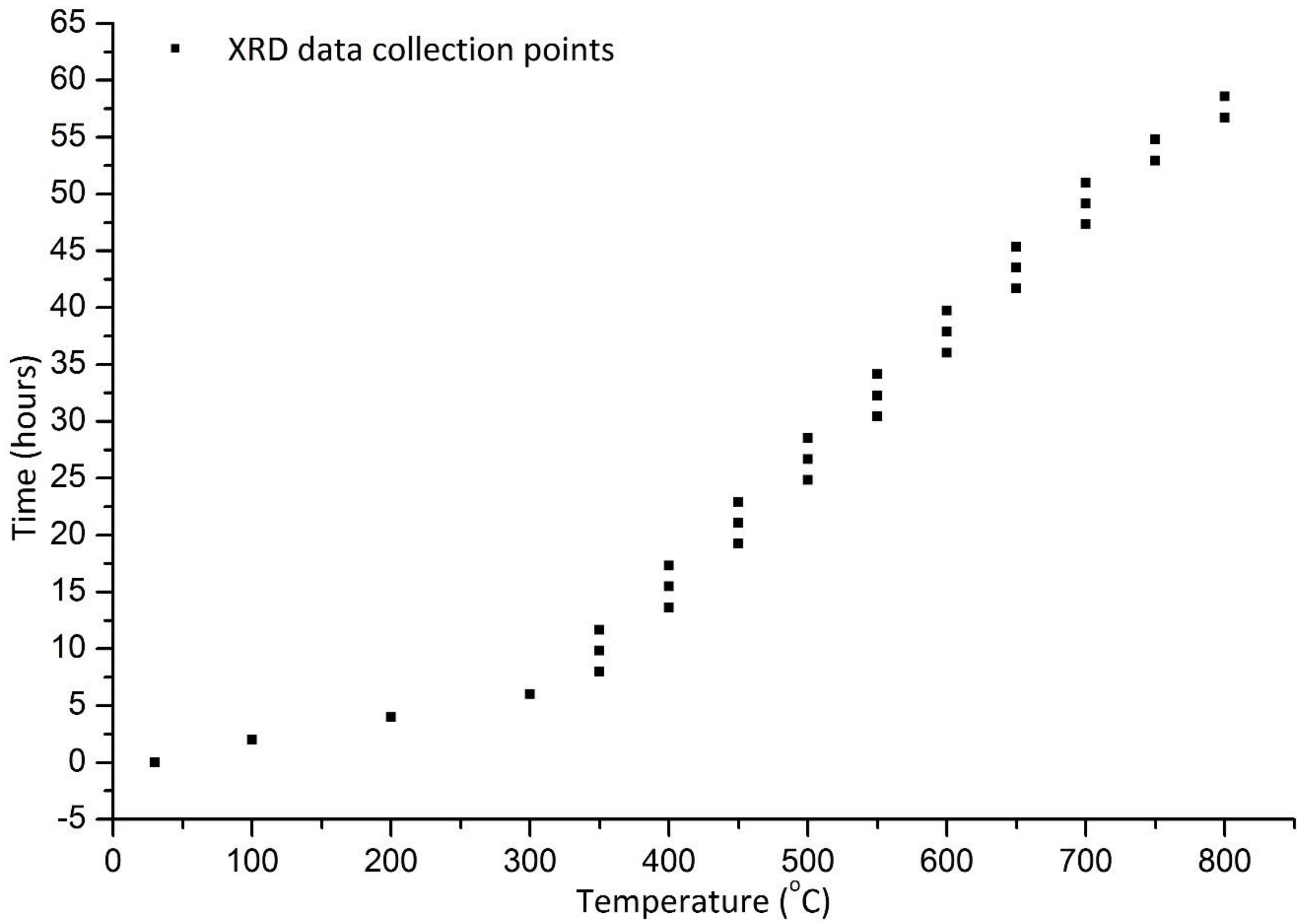

Figure S2: Plot of temperature vs time for the *in-situ* PXRD data collection.

Single patterns were collected at 100°C, 200 ^{o}C and 300°C followed my multiple patterns per temperature from 350°C up to 810°C. *In-situ* diffractograms are shown in Figure S3 with selected Rietveld refinements shown in Figures S3 (A-D) during the phase transformation from anatase to rutile. Refinements were performed on all data and phase percentages of anatase and rutile are graphed in Figure S4.

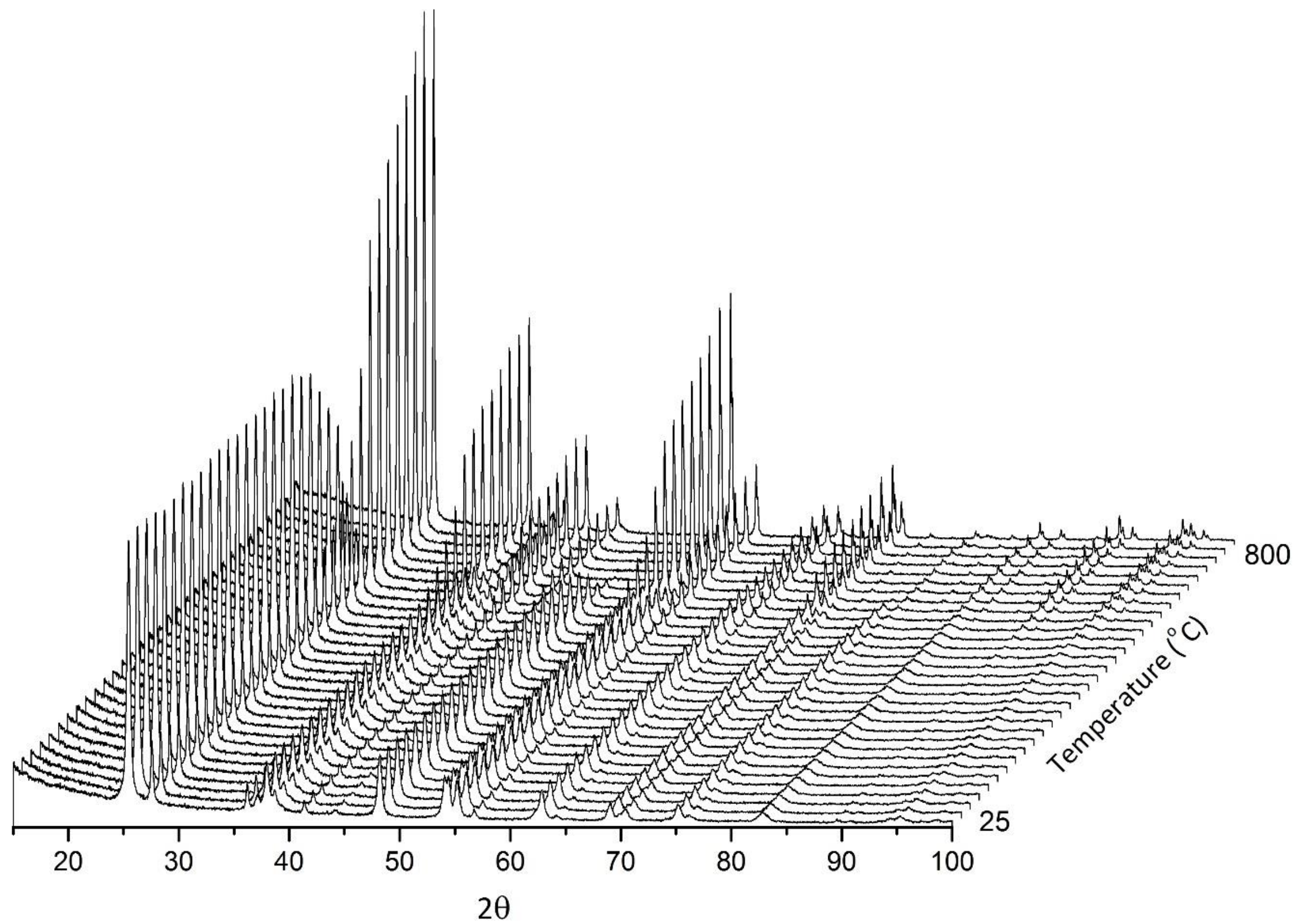

Figure S3: *In-situ* diffraction patterns collected from ambient temperature to 800°C of commercial Aurolite® (1% Au-P25). Most noticeably in the Figure S3 is the phase change from anatase to the thermodynamically stable rutile phase during the thermal treatment. Examples of the difference patterns attained from Rietveld refinements are shown in Figure S4 (A-D) taken from the refinements performed on data collected at 650°C.

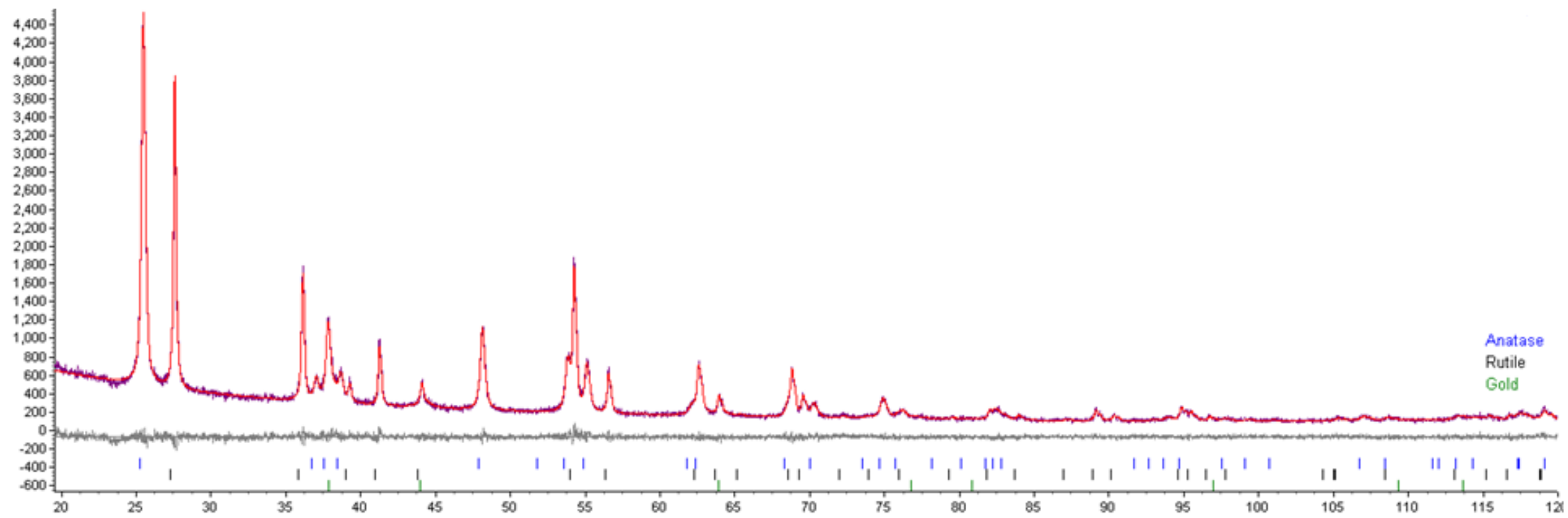

Figure S4-A: Difference pattern from Rietveld refinement of a diffraction pattern collected during the *in-situ* measurement at 650°C.

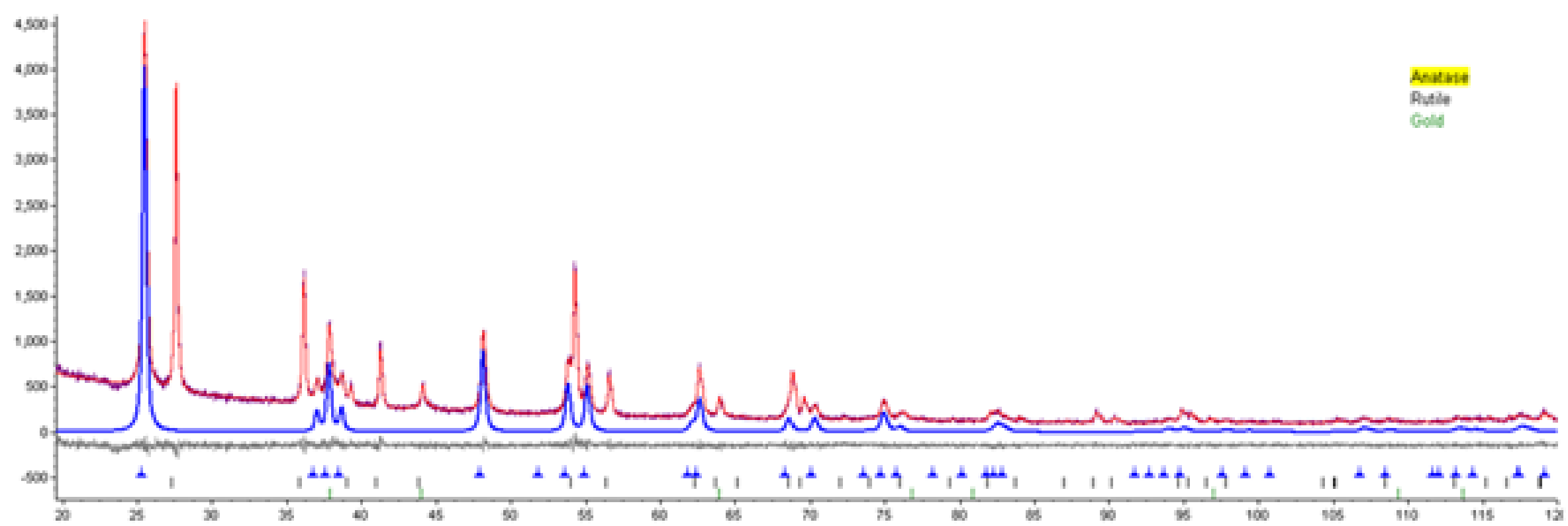

Figure S4-B: Difference pattern highlighting the anatase phase in blue.

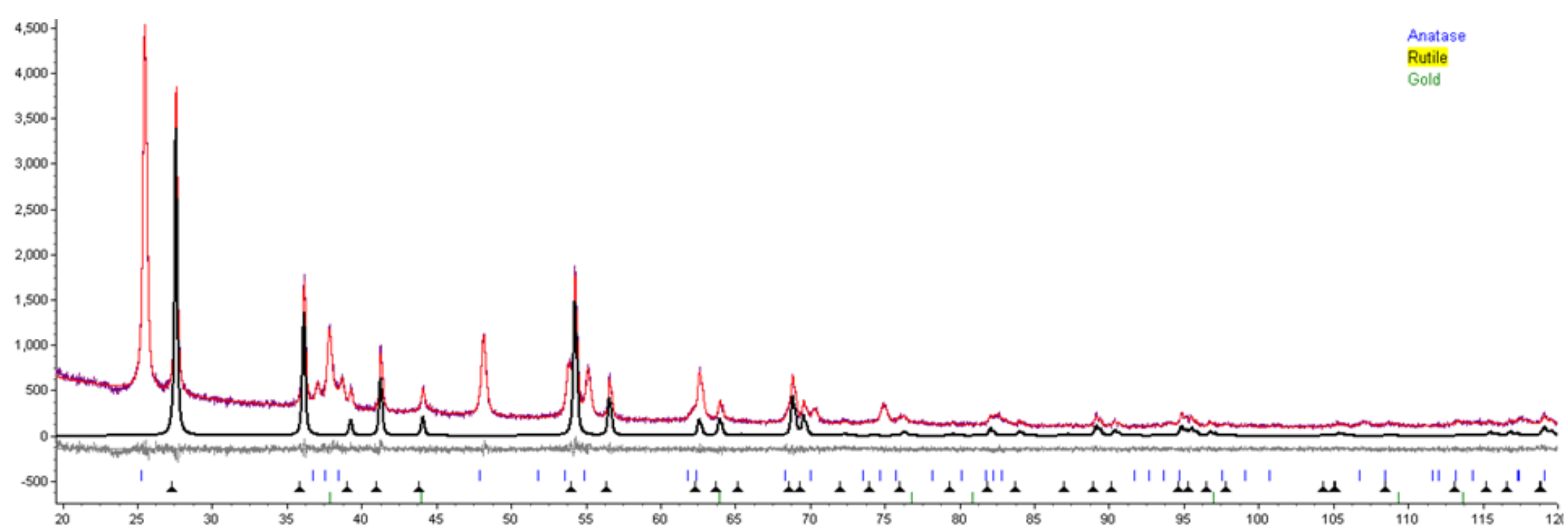

Figure S4-C: Difference pattern highlighting the rutile phase in black.

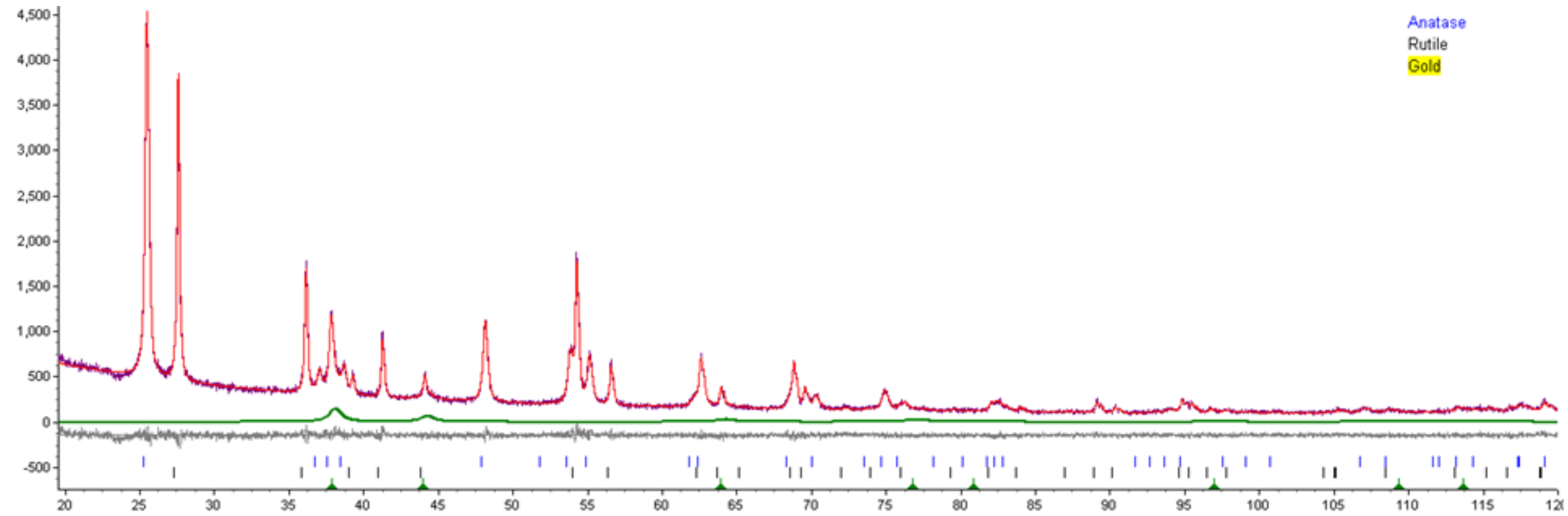

Figure S4-D: Difference pattern highlighting the Au phase in green.

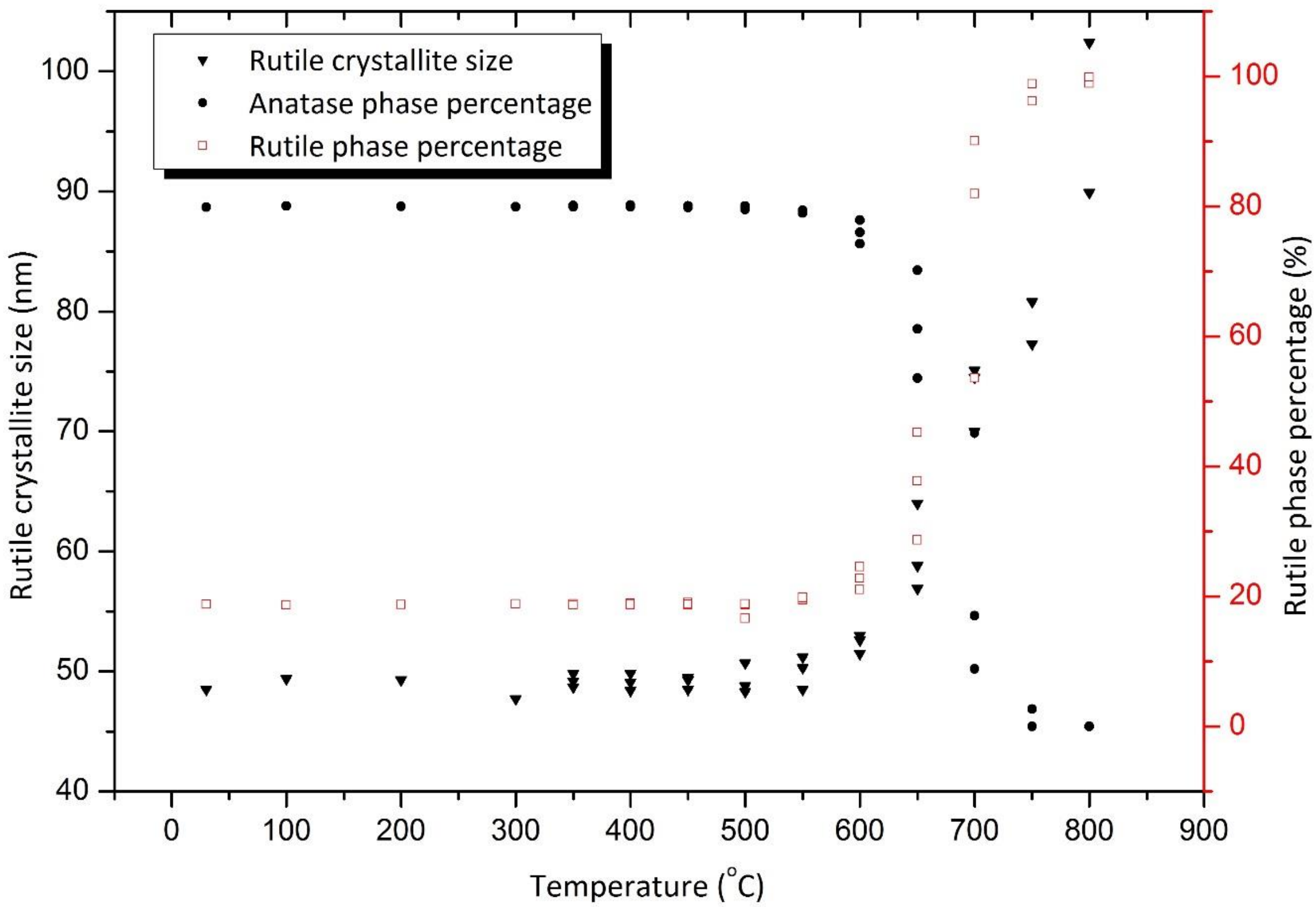

Figure S5: Rietveld analysis results showing phase composition as well as crystallite sizes of the anatase and rutile crystallites with respect to temperature.

Initially, the catalyst comprises around 20% rutile phase and 80% anatase. The crystallite size the rutile phase is approximately 50 nm up to 500°C. Above 550°C however, their growth takes on an exponential trend. The data shows the crystallite size increase is correlated to the phase transformation of both TiO_2 polymorphs with the largest increases observed during the phase transformation as the anatase is consumed to form rutile. At the given heating rate with respect to time, the phase transition was complete around 750°C. From 600°C to 700°C the rutile crystallite size changes from around 50 nm to 80 nm and finally to over 100 nm by 800°C. Surface areas of 47 m^2/g for the Aurolite® catalyst prior to thermal exposure determined by BET. After thermal exposure, the surface area had decreased substantially to 7 m^2g. The 7 fold decrease in surface area is attributed to both

the sintering of the support phase particles, but more notably to the anatase to rutile phase transformation. As the usable surface area is an important parameter for a catalyst support, this substantial loss of surface area indicates reasons as to why catalytic performance is lost at 500 and 800°C. The refinement results for the Au and rutile crystallite sizes are shown in Figure S6 and demonstrate why the Aurolite® catalyst is so efficient at low to medium temperature ranges from ambient to 450°C.

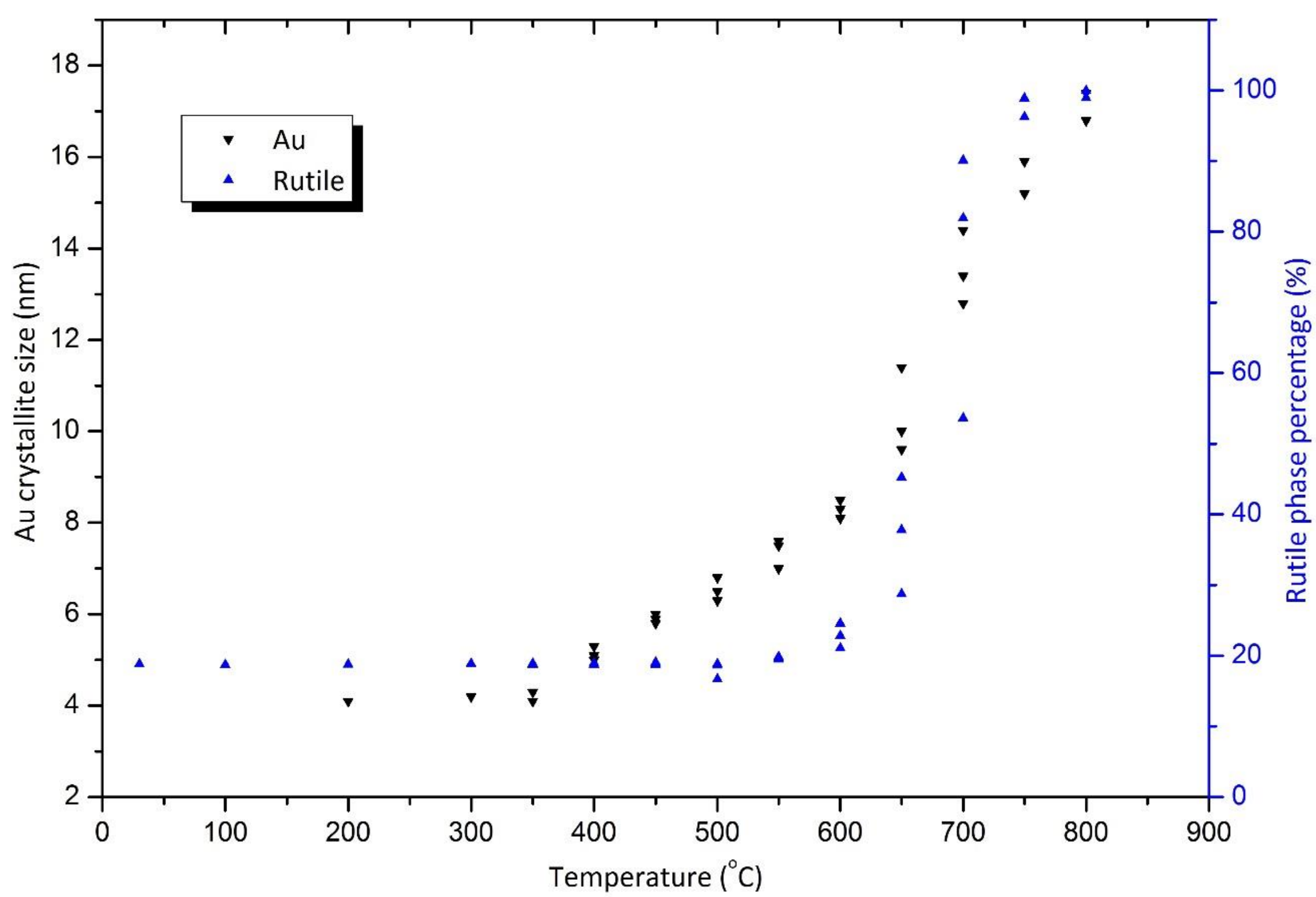

Figure S6: Rietveld results showing the rutile phase percentage and Au crystallite size versus temperature. At ambient to moderately high temperatures the catalyst maintains stable Au and support particle sizes. However, above 550°C, the catalyst undergoes major structural changes where increases in the rutile polymorph (phase transition) coincide with large increases in Au crystallite size.

From ambient to 450°C the Au crystallites are small and hence able to readily facilitate the oxidation of CO. As heating continues the commencement of a large increase in Au crystallite size can be observed especially at temperatures above 550°C. The rutile phase composition and Au crystallite size show correlated growth trends where the most significant Au sintering occurs during the phase change as anatase is consumed to form rutile. From 550°C to 700°C the Au size more than doubles in size over only a 150°C temperature change. The large change in Au crystallite size correlates well to what is observed in the catalytic results (Figure S1) with active catalysts noted at lower temperatures but inactive catalysts after thermal treatment to 700°C. The conversion of anatase to rutile occurs through a wave-front progression in the anatase crystal. Along this front, the titanium-oxygen octahedra rearrange through a rotation process. The front extends to the surfaces of the support particles. Thus, it is likely that Au nanoparticles on the surface of the anatase would be caught by the front migration and be moved along with the wave-front. This would promote sintering of the Au as the nanoparticles would be moved into closer proximity as well as likely encapsulation of the active sites.

Au nanoparticle sizes of 19 nm were determined from PXRD data collected after the catalytic reaction as shown in Figure S7, correlating well with the CO oxidation results as Au particles of this size will not facilitate low-temperature CO oxidation. Metallic nanoparticles on the surface of various metal oxide supports have been shown to increase the rate of phase conversion at lower temperatures.[1-2] Relatively short exposures of Aurolite® and other current Au-TiO_2 based catalysts may be able to withstand elevated temperatures for short durations but once phase transformations occur the catalyst deactivates as seen by the catalytic testing. Figure S7 shows the catalyst after thermal

treatment with all anatase converted to rutile and the presence of a distinct Au peak at 38.2° 2θ.

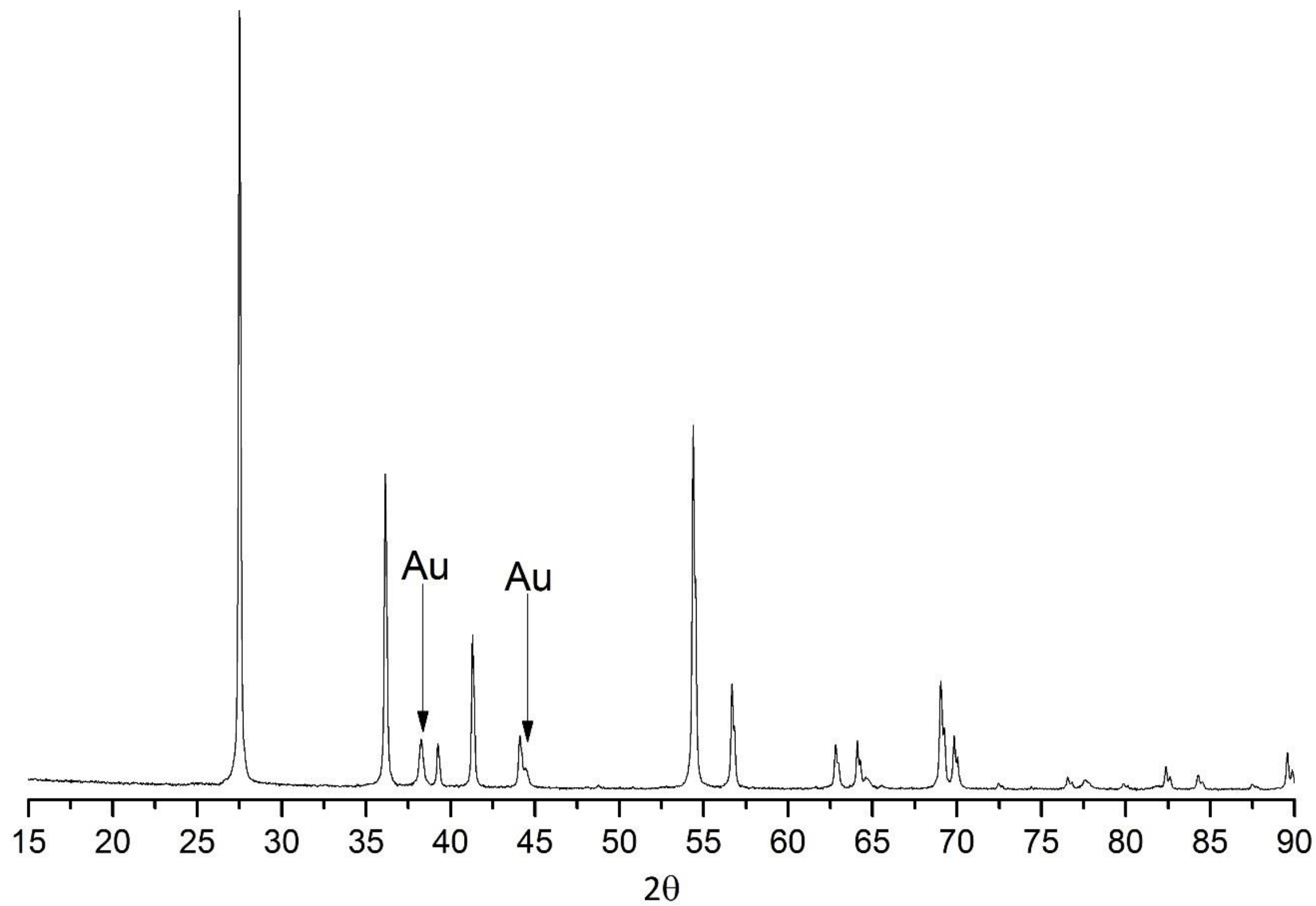

Figure S7: Commercial Au-TiO_2 after catalytic reaction showing a complete phase transition to rutile phase TiO_2 with Au peaks corresponding to crystallite sizes of approximately 19 nm.

Phase changes are inevitable as the thermodynamically more stable product is favoured, the only factor is how rapidly the transformation occurs and thus how rapidly deactivation occurs. This conversion resulted in significant loss of surface area as well as sintering and encapsulation of Au nanoparticles resulting in an unstable catalyst at high temperatures.

TEM

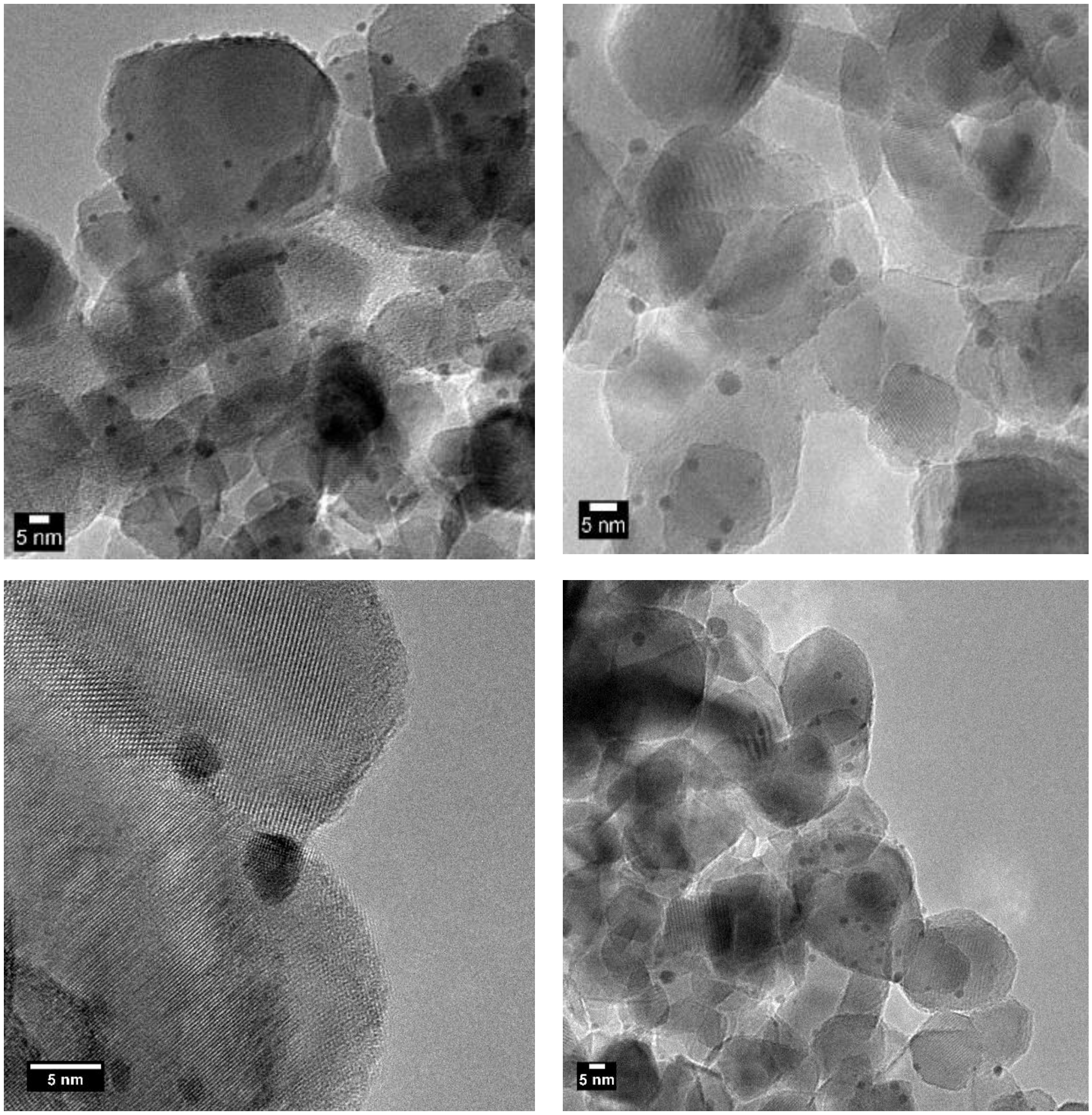

Figure S8: TEM of commercial Au-TiO_2 catalysts after calcination at 300^oC.

Au-RANR catalysts

***In-situ* PXRD**

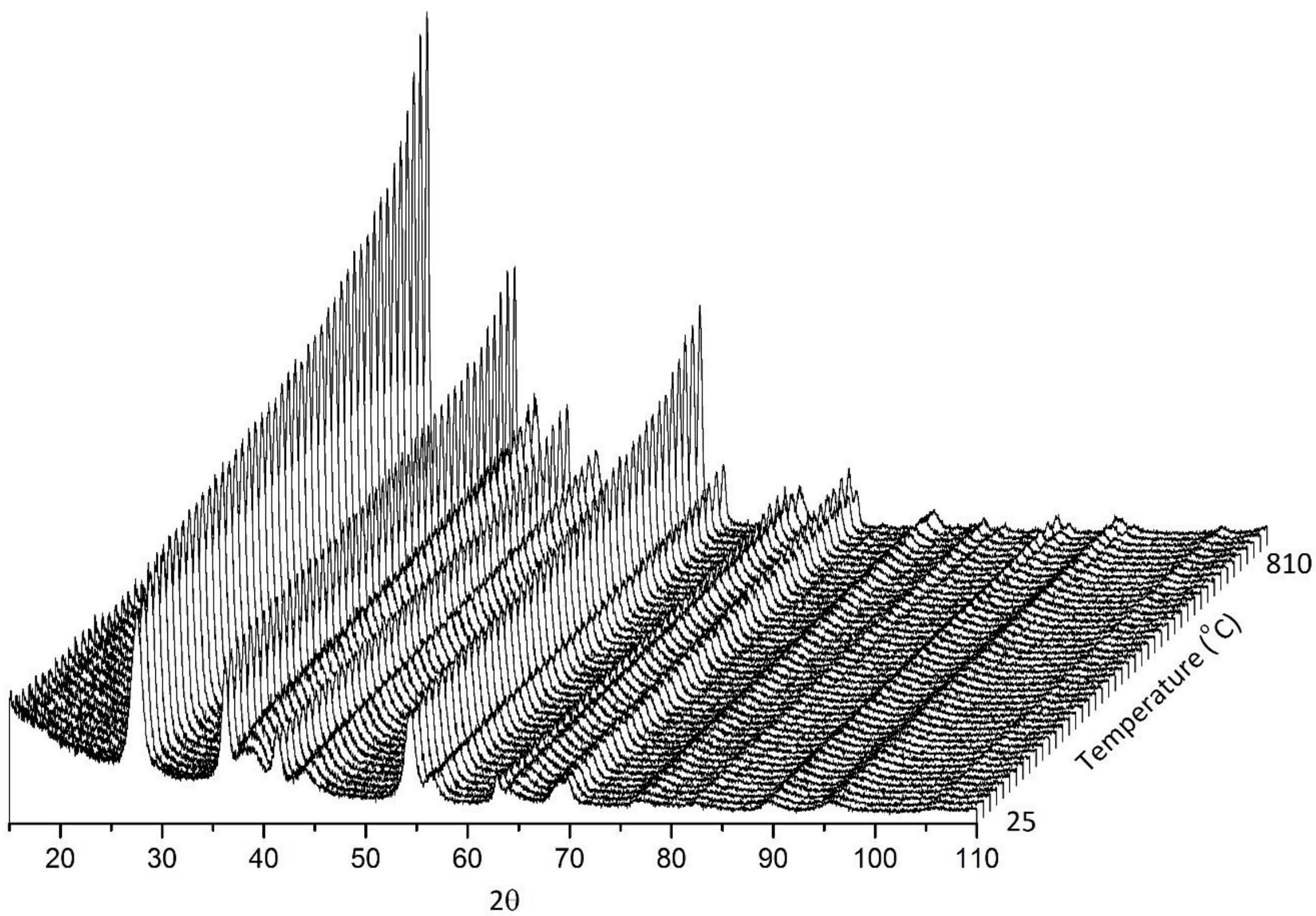

Figure S9: *In-situ* diffractograms of 5% Au-RANR. Note only every second pattern is shown here for clarity of the image.

Rietveld refinements were performed on all collected patterns to determine changes in the crystallite sizes during thermal treatment. An example of a Rietveld refinement after the catalytic reaction is shown below in Figure S10 (A-C).

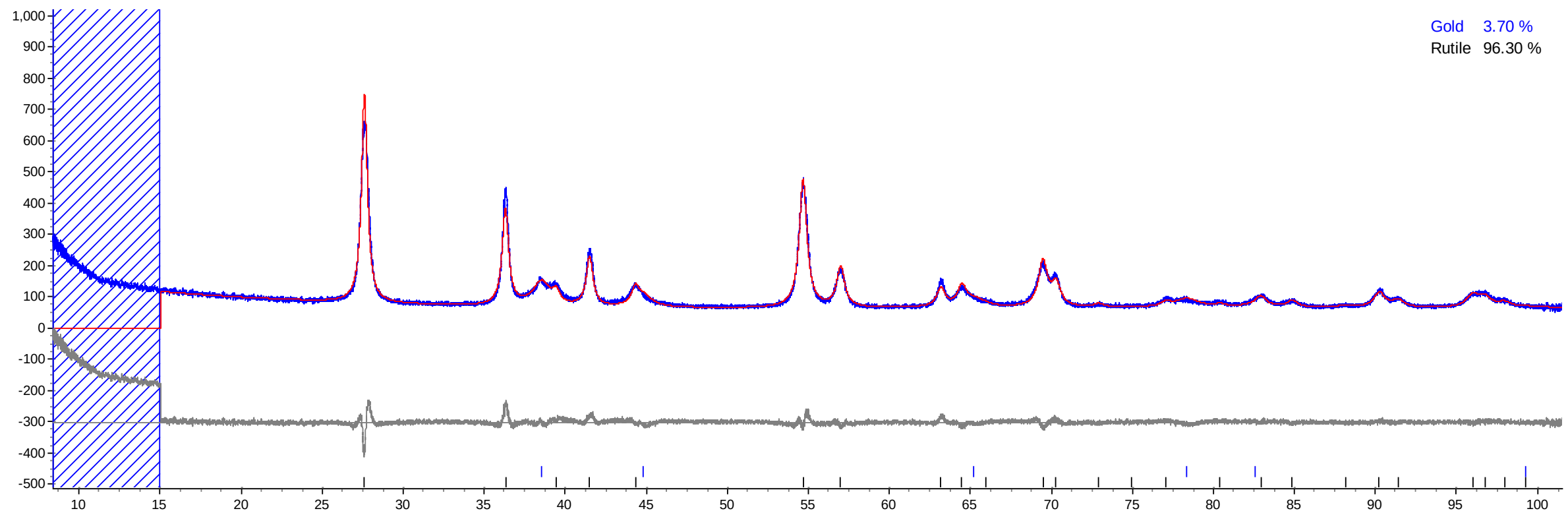

Figure S10-A: Difference patterns of Rietveld refinements performed on diffractograms collected after the catalytic reaction.

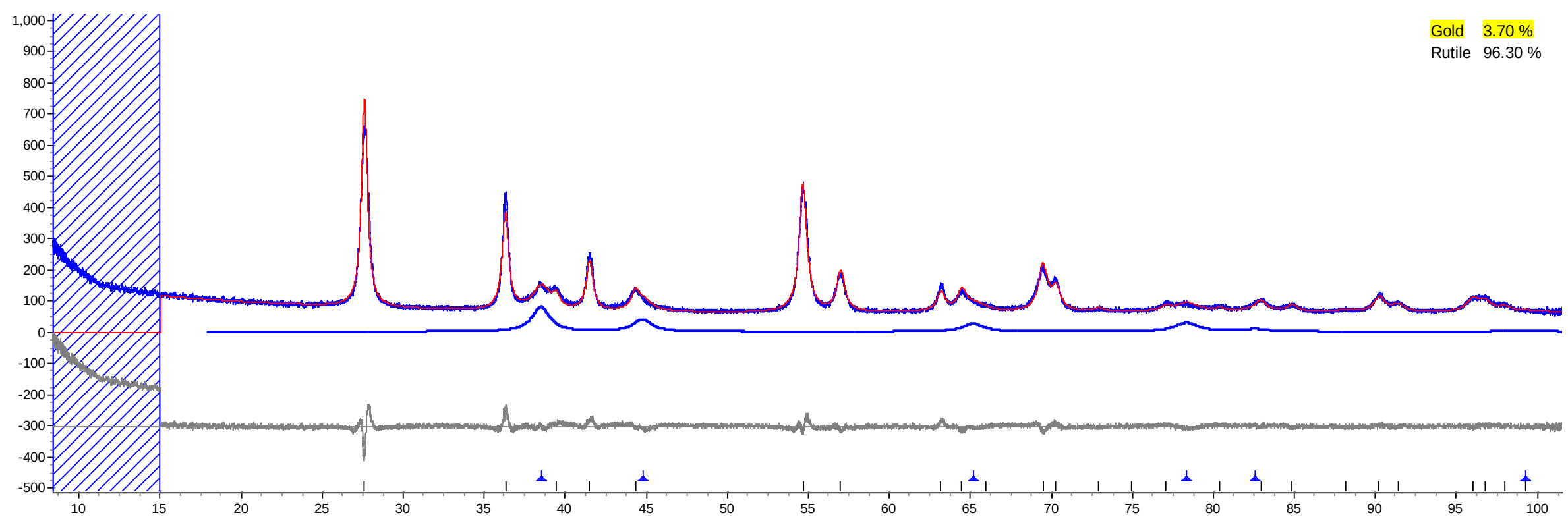

Figure S10-B: Difference patterns of Rietveld refinements highlighting the Au phase.

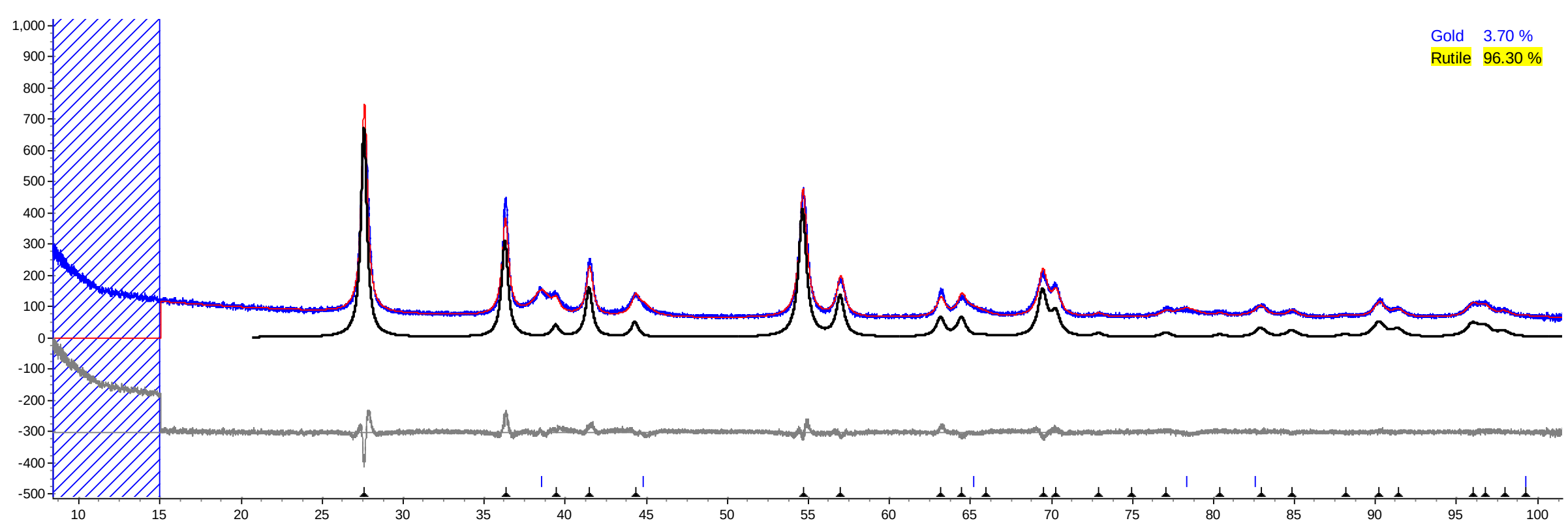

Figure S10-C: Difference patterns of Rietveld refinements highlighting the rutile phase.

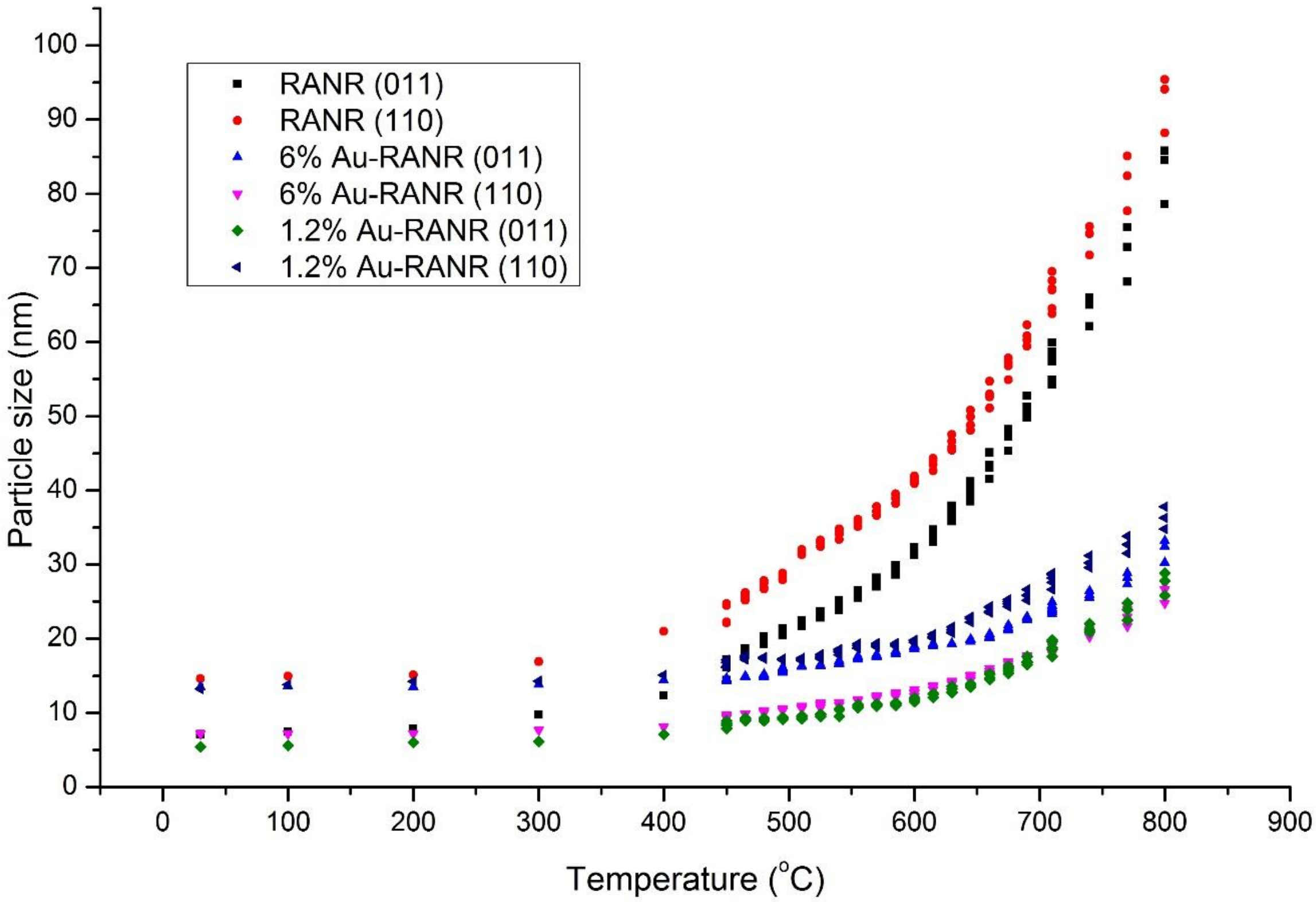

Figure S11: Comparisons of the growth of the RANR structure along (0 1 1) and (1 1 0) planes with respect to temperature with no Au, 1.2% Au and 6% Au. The presence of Au resulted in a stabilizing effect with regards to the growth of the support structure.

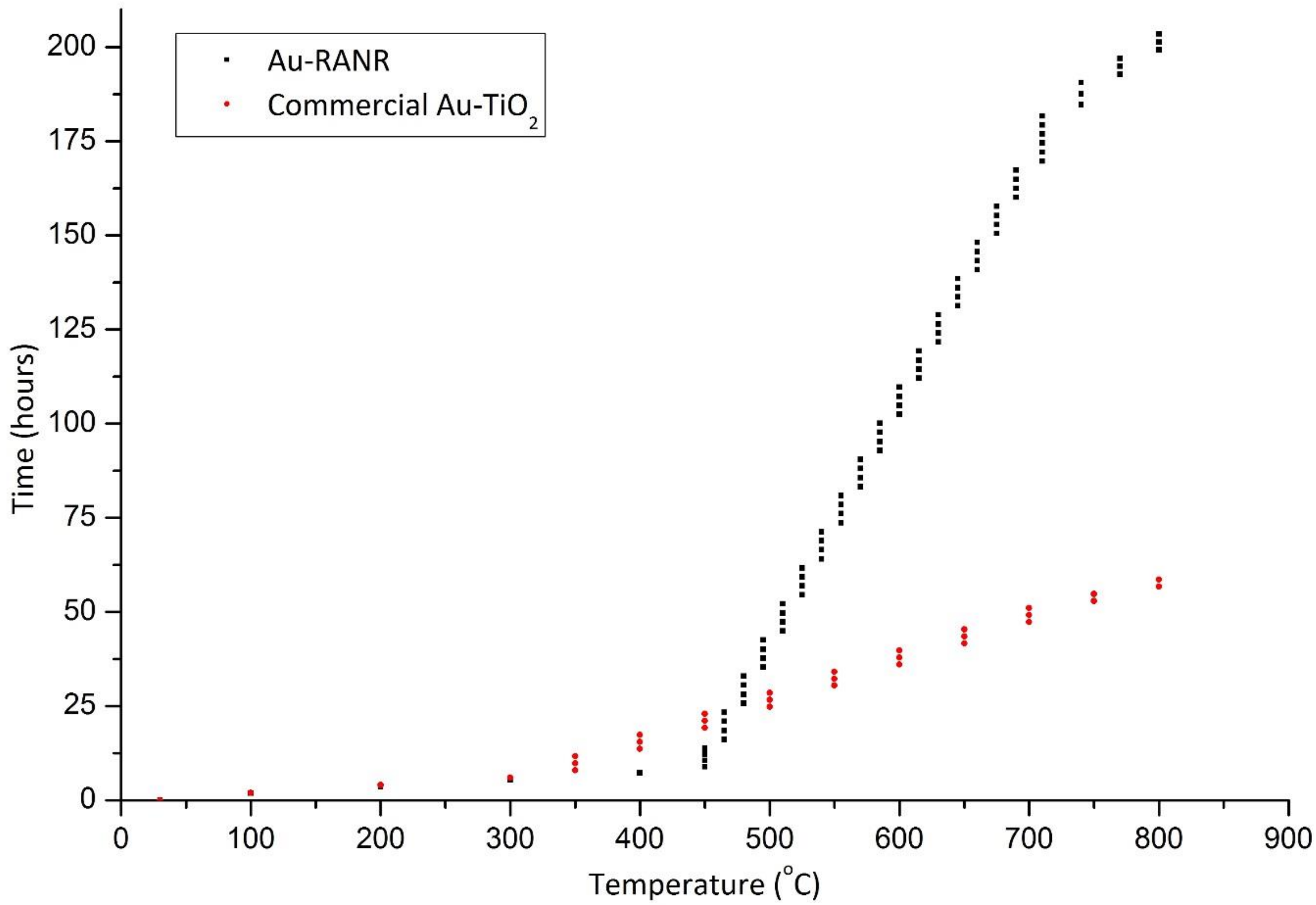

Figure S12: Commercial Au-TiO_2 *in-situ* PXRD collection time vs Au-RANR data collection times. *In-situ* PXRD was conducted for longer durations (in excess of 200 h) compared to the commercial Au catalysts as shown in figure S3.

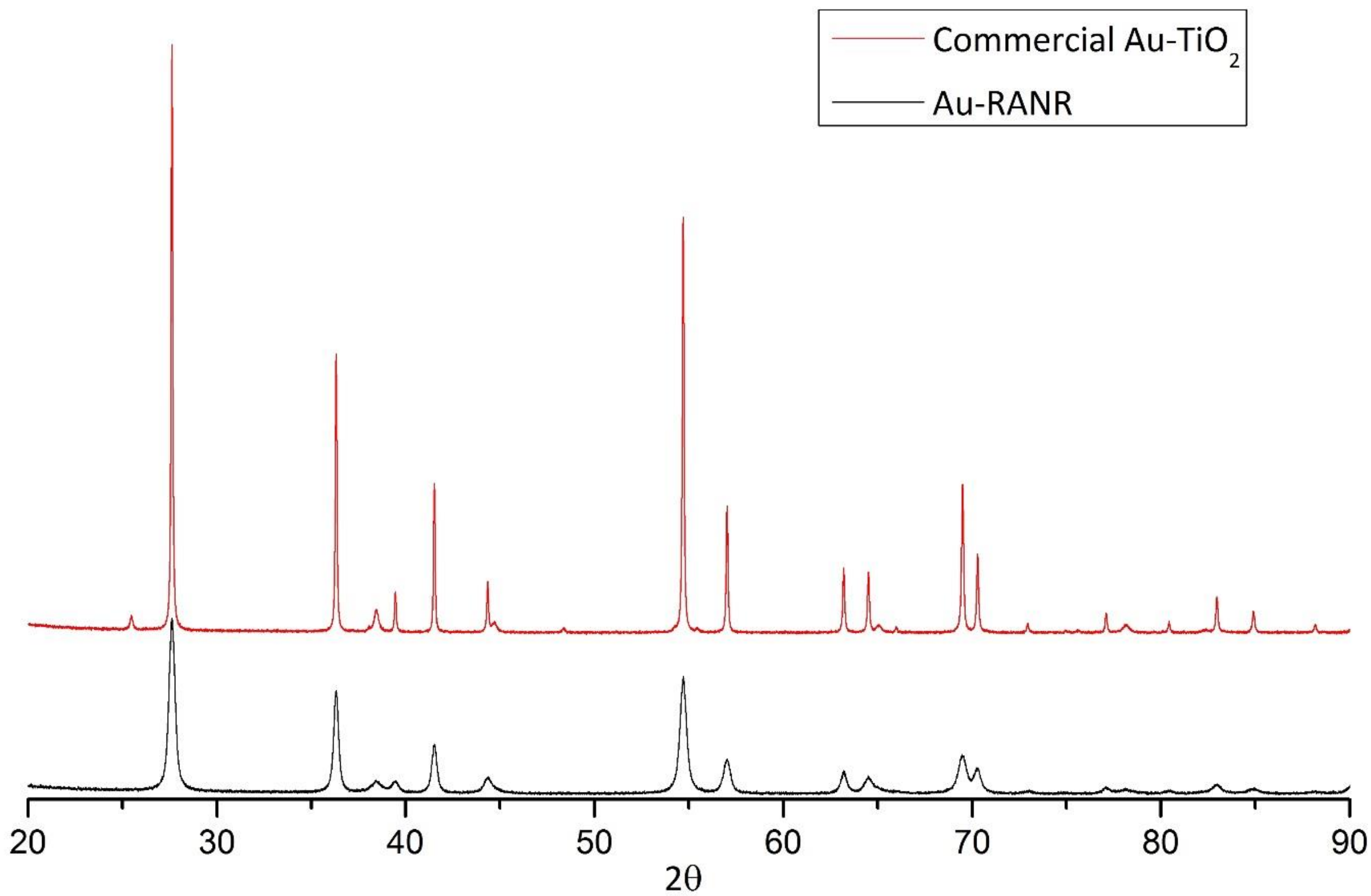

Figure S13: 1% Au loadings on commercial and RANR after the catalytic reaction.

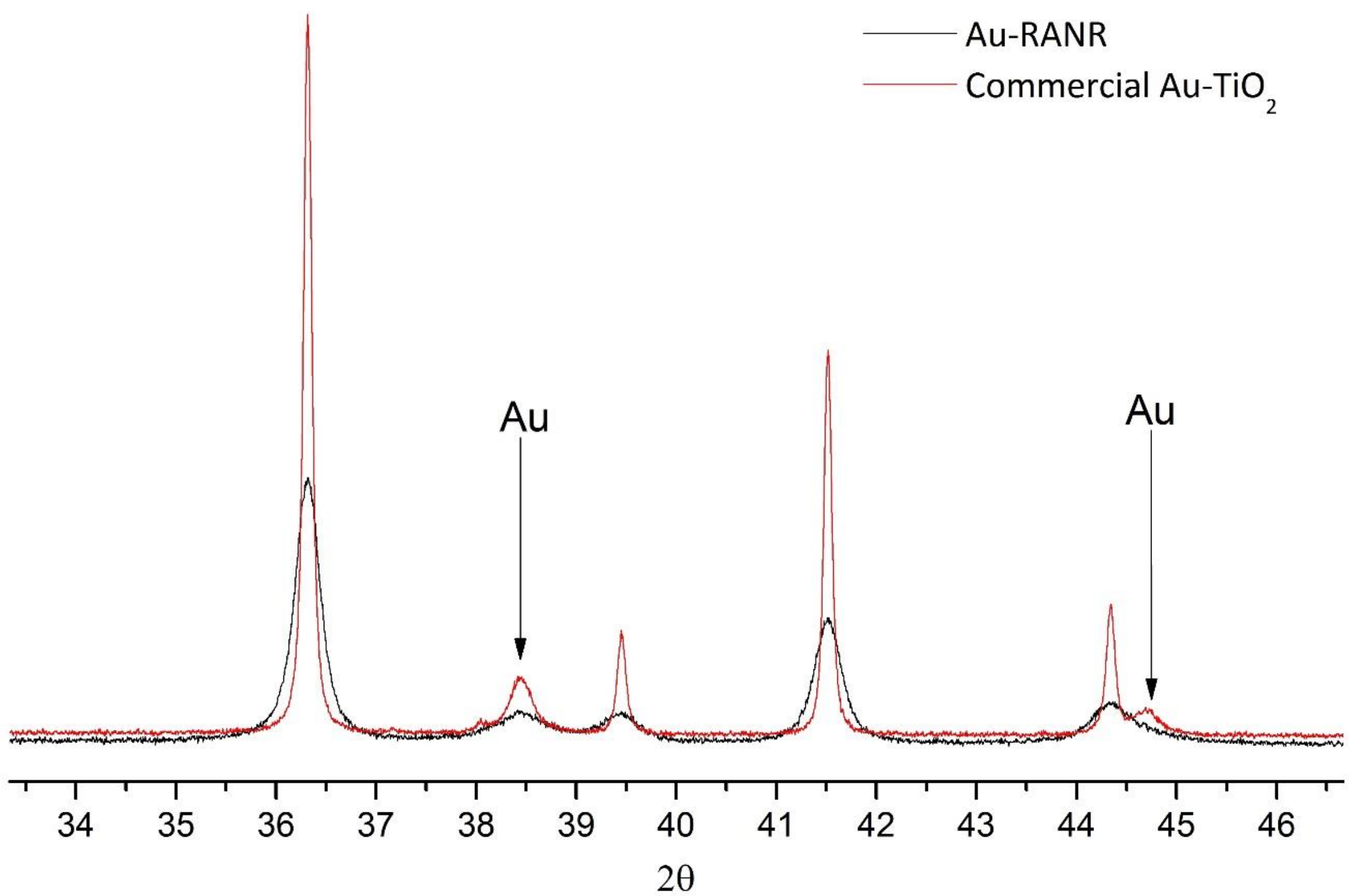

Figure S14: Zoomed view of comparing Au peaks from 1% commercial Au-TiO_2 and 1% Au-RANR catalysts after thermal cycling to 800°C. Sharp diffraction (small FWHM) peaks for the commercial Au-TiO_2 catalyst show large, sintered Au and support crystallites now composed of rutile phase due to phase conversion of the support while the Au-RANR maintains its small Au and nano-support characteristics.

TEM

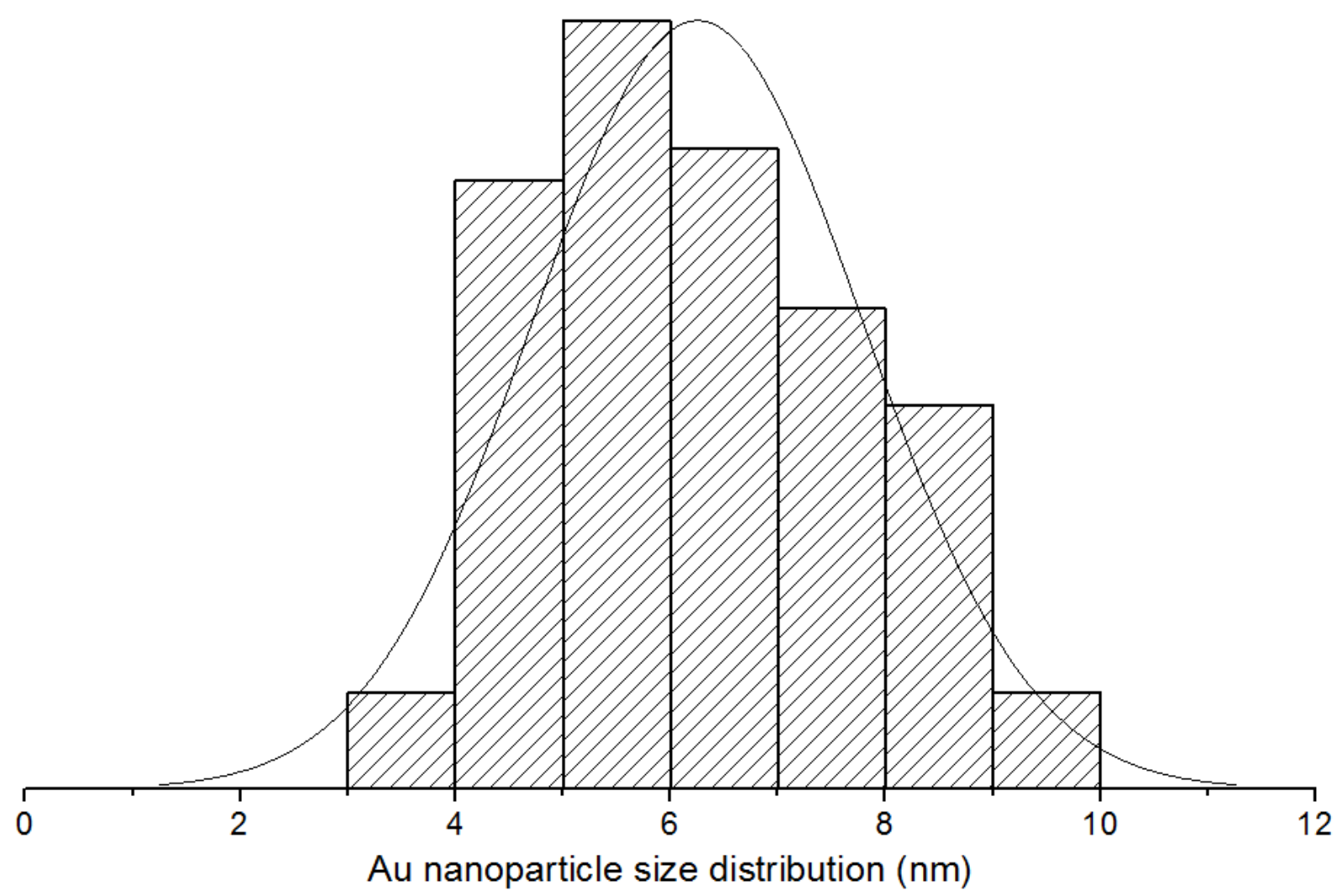

Figure S15: TEM distribution of nanoparticle of 6% Au-RANR after thermal treatment taken from sampling over 300 particles.

Pair-Distribution function (PDF) analysis

Pair Distribution Function analysis was conducted to complement PXRD and TEM results as well as gain further understanding of the catalysts behavior during thermal treatments. PDF considers both the Bragg and diffuse scattering from the sample and is thus very sensitive to disordered materials, including nanocrystalline and amorphous materials. Au-RANR catalyst with 6% nominal Au loading was analyzed *in-situ* from 25°C to 600°C and changes in the structural parameters were quantified.

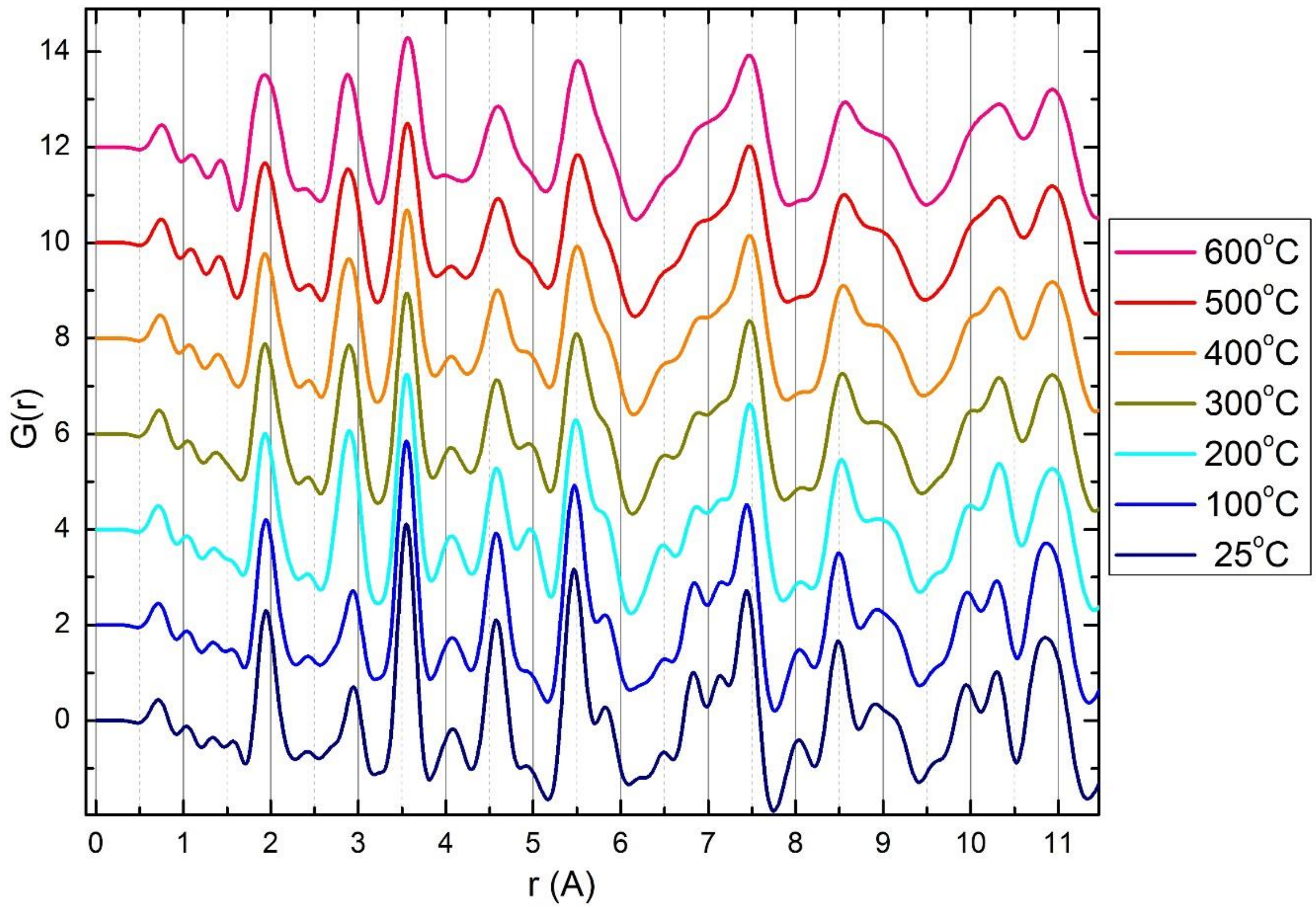

Figure S16: Pair distribution function measurements with temperature from 25 to 600°C, corresponding to the rutile support and the Au nanoparticles (main Au peak at approx. 2.88 A).

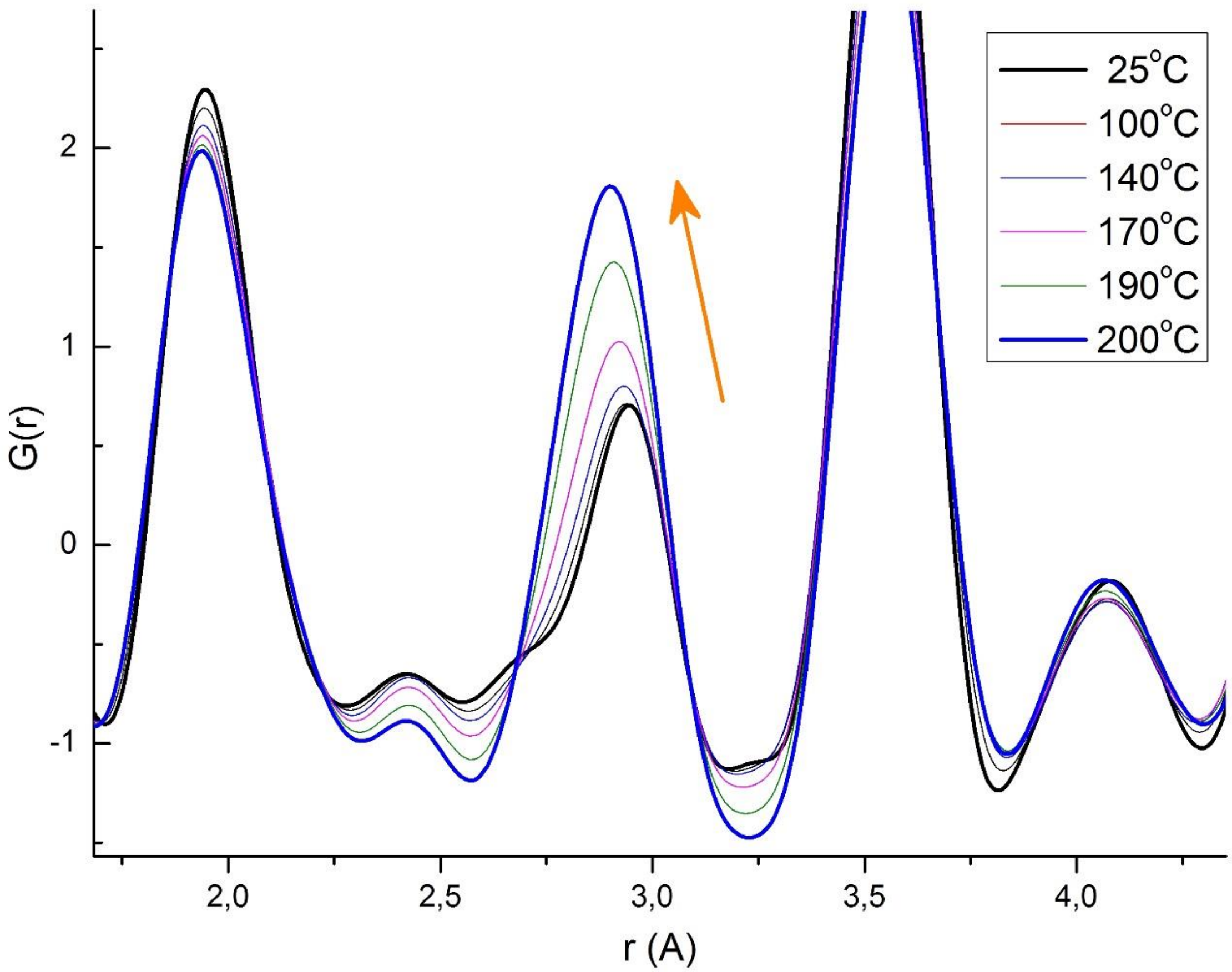

Figure S17: Formation of the Au peak of the nearest neighbour atoms during the initial heating from ambient temperature to 200°C. This corresponds to the reduction of Au (III) to metallic Au in the nanoparticles. It is known that the reduction temperature depends on the support, being 200°C for silica and 130°C for anatase supports. Our results indicate a full Au reduction at 200°C for the rutile support. The Au phase fraction increased strongly from 25 to 200°C, while the reduction of Au (III) was taking place. Above 200°C, the Au phase fraction varied weakly, with an average value of 5.4%.

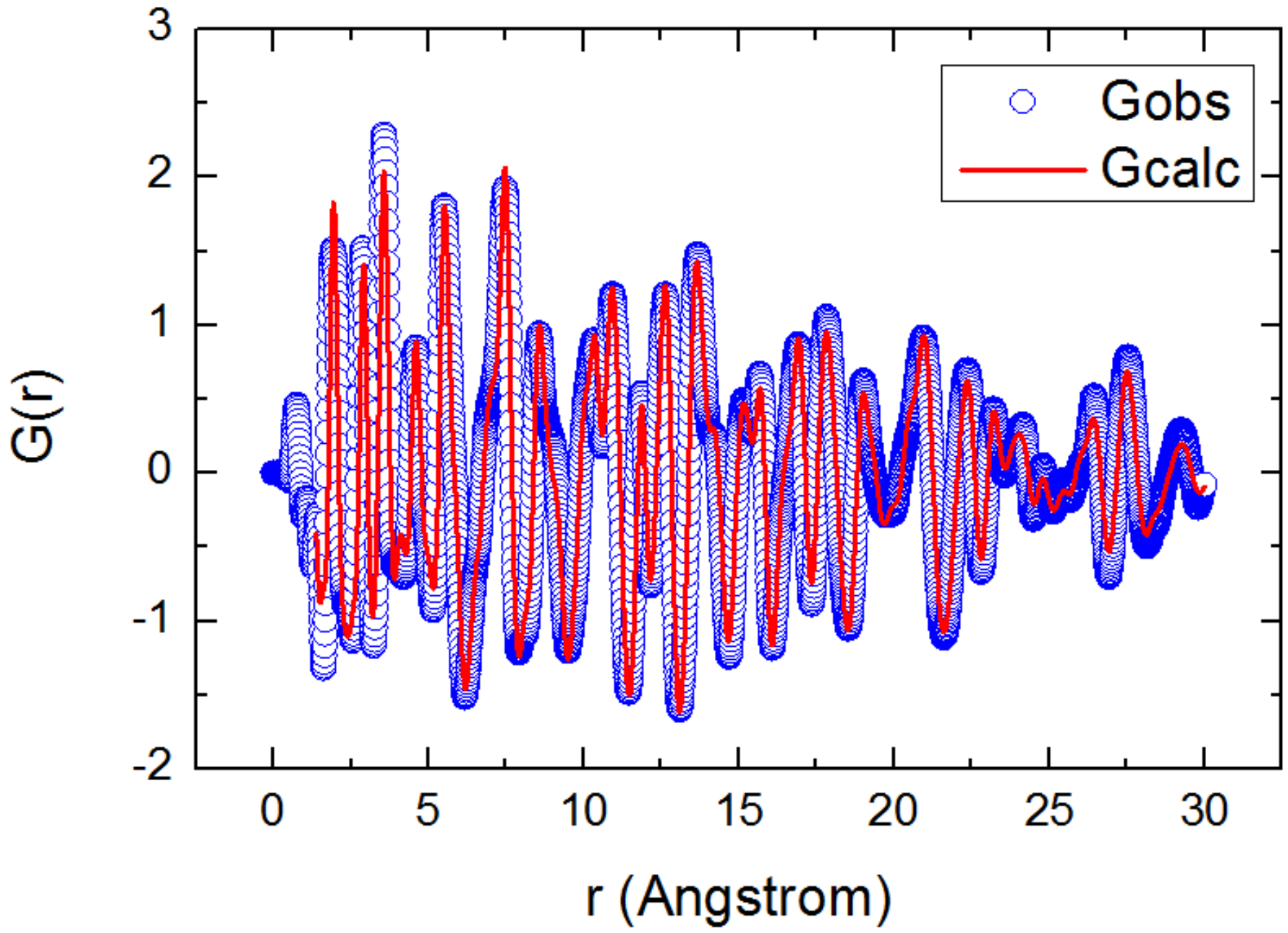

Figure S18: The PDF measurements were simulated with a model including rutile and Au phases. The fitting parameters were: phase fractions for rutile and Au, particle size (spherical model), lattice parameters, peak width correction for correlated motion, atomic displacement parameters, and oxygen fractional coordinates. Parameters for instrument resolution were taken from the fit of CeO_2 standard material. The fits to the measurements confirmed the phase purity of the rutile support, as concluded also from the *in-situ* XRD analysis.

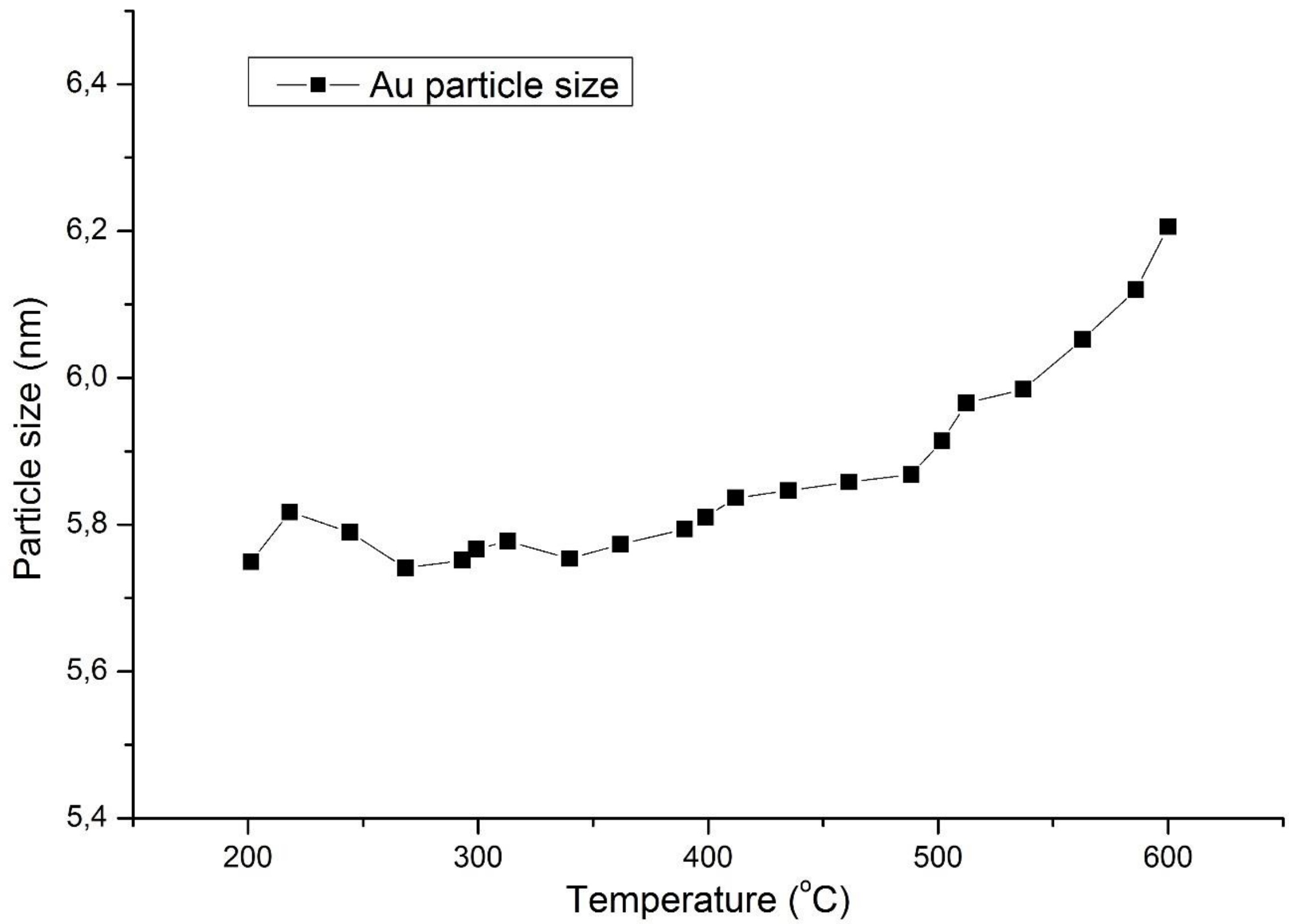

Figure S19: Au particle size as a function of temperature obtained from the simulation of the PDF measurements. A spherical model was considered during the simulations. Even though the metal loading differed between *in-situ* XRD and PDF samples the Au particle sizes still remained within close proximity of each other showing that the catalyst is able to maintain small Au particle sizes over a range of Au loadings (from 1% to 6%). The PDF measurements once again confirmed the stability of the catalyst with a change of only 0.5 nm over the temperature range for the Au nanoparticles.

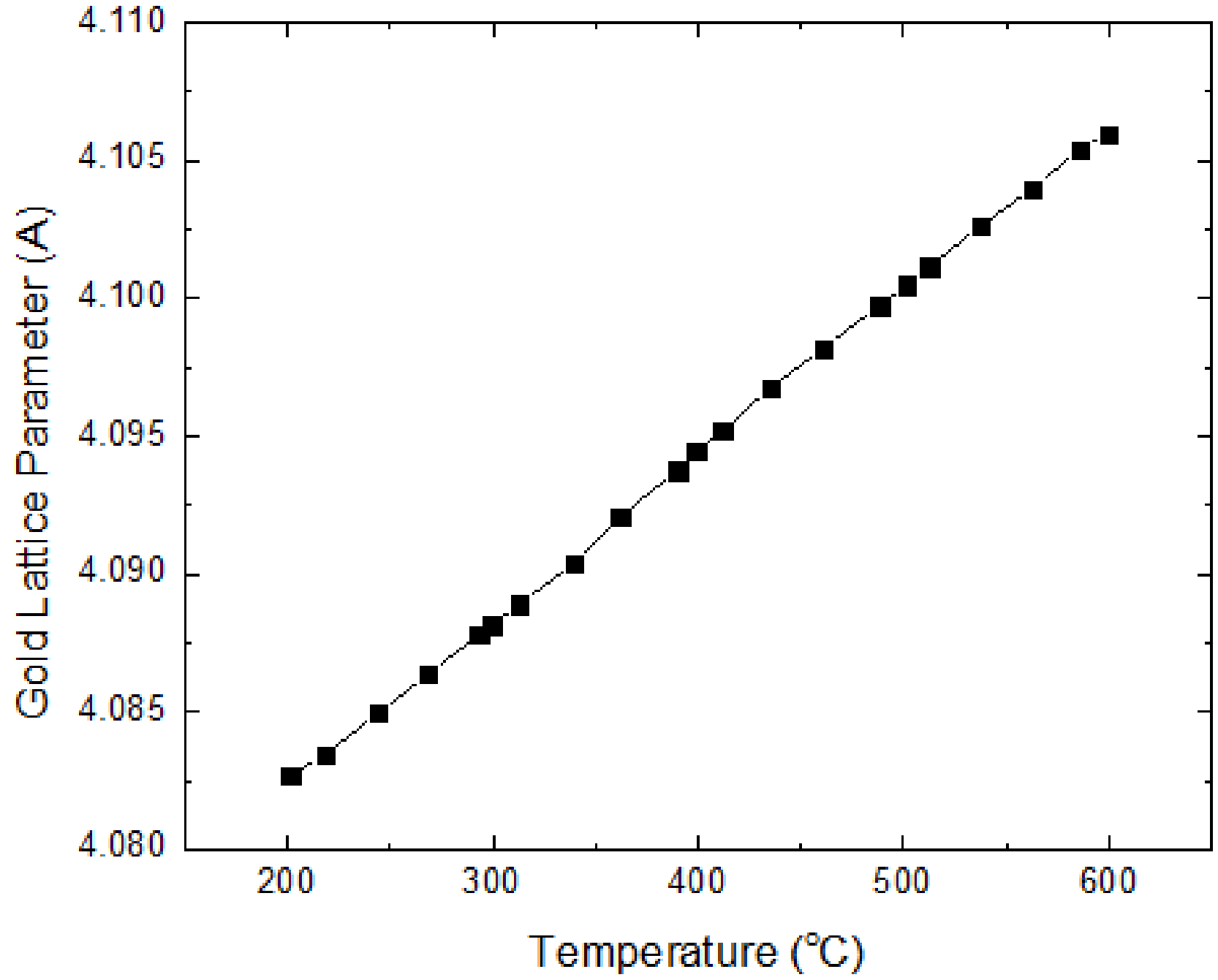

Figure S20: Au lattice parameter versus temperature.

The fitted Au lattice parameter is slightly smaller than bulk values for all temperatures. This agrees with previous results for Au nanostructures.[3]

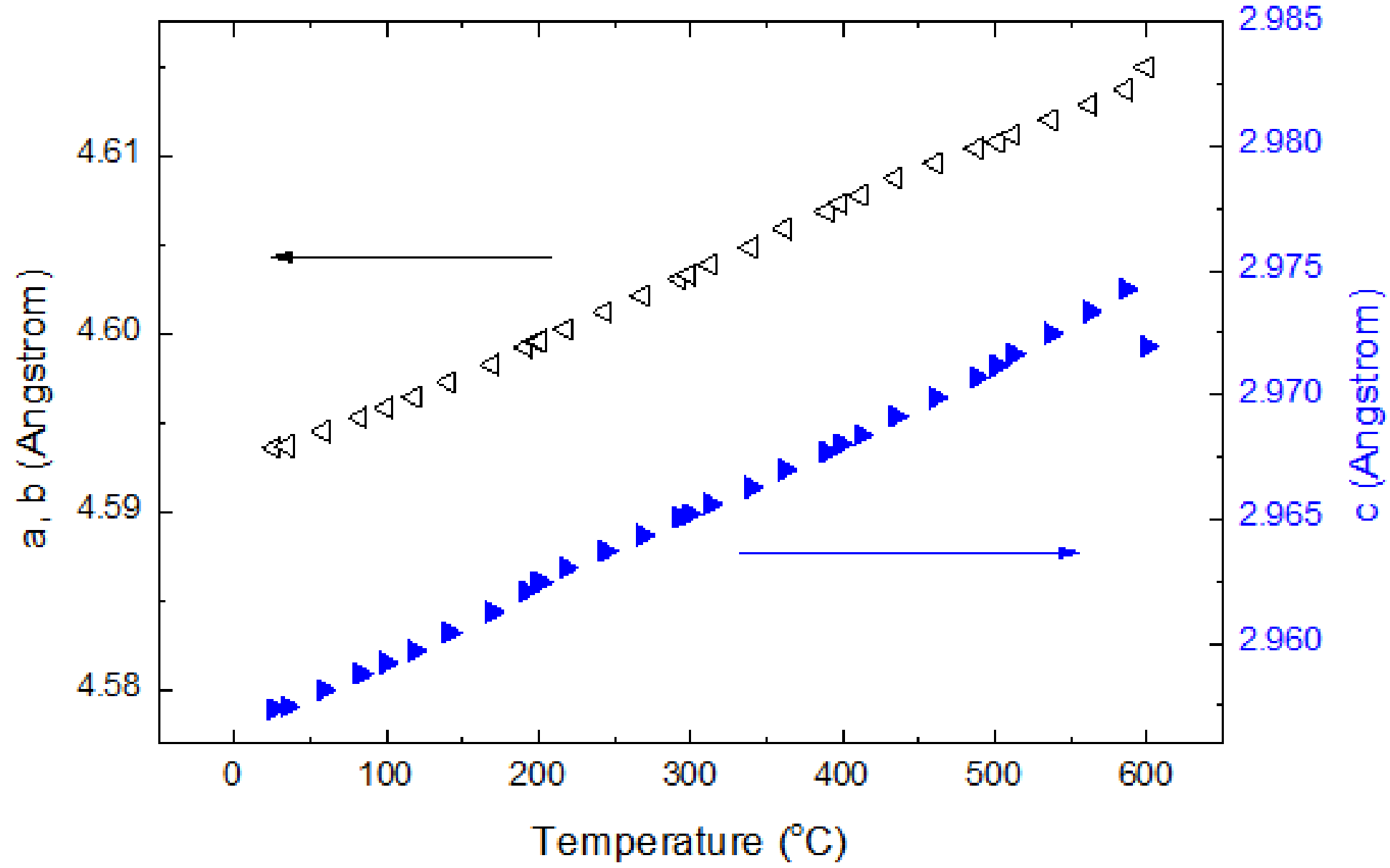

Figure S21: Rutile support lattice parameter dependence with temperature, very close to the bulk.

Acknowledgements

Erico T. Neto for SEM analysis conducted at the Brazilian Nanotechnology National Laboratory, Brazilian Center for Research in Energy and Materials, Rua Giuseppe Maximo Scolfaro, 10.000, Campinas, SP 13085-903, Brazil, LNNano.

FAPESP project: **2012/17942-6**

References

1. Q. Xu, J. Zhang, Z. Feng, Y. Ma, X. Wang and C. Li, Surface Structural Transformation and the Phase Transition Kinetics of Brookite TiO2, *Chem.–Asian J.*, 2010, **5**, 2158.
2. J. F. Ban・eld, B. L. Bischoff and M. A. Anderson, Thermal Stability of Brookite – TiO2 Nanoparticles with Controlled Size and Shape: In situ Studies by XRD, *Chem. Geol.*, 1993, **110**, 211.
3. V. Petkov, Y. Ren, S. Shan, J. Luo and C. J. Zhong, A distinct atomic structure–catalytic activity relationship in 3–10 nm supported Au particles, *Nanoscale*, 2014, **6**, 532.
